# Application of a Highly Selective Cathepsin S Two-step Activity-Based Probe in Multicolor Bio-Orthogonal Correlative Light-Electron Microscopy

**DOI:** 10.3389/fchem.2020.628433

**Published:** 2021-02-08

**Authors:** Floris J. van Dalen, Thomas Bakkum, Tyrza van Leeuwen, Mirjam Groenewold, Edgar Deu, Abraham J. Koster, Sander I. van Kasteren, Martijn Verdoes

**Affiliations:** ^1^ Department of Tumor Immunology and the Institute for Chemical Immunology, Radboud Institute for Molecular Life Sciences, Radboud University Medical Centre, Nijmegen, Netherlands; ^2^ Leiden Institute of Chemistry and the Institute for Chemical Immunology, Leiden University, Leiden, Netherlands; ^3^ Chemical Biology Approaches to Malaria Laboratory, The Francis Crick Institute, London, United Kingdom; ^4^ Department of Cell and Chemical Biology, Leiden University Medical Center, Leiden, Netherlands

**Keywords:** cathepsin S, two-step activity-based probe, bio-orthogonal labeling, correlative light-electron microscopy, cathepsin activity localization

## Abstract

Cathepsin S is a lysosomal cysteine protease highly expressed in immune cells such as dendritic cells, B cells and macrophages. Its functions include extracellular matrix breakdown and cleavage of cell adhesion molecules to facilitate immune cell motility, as well as cleavage of the invariant chain during maturation of major histocompatibility complex II. The identification of these diverse specific functions has brought the challenge of delineating cathepsin S activity with great spatial precision, relative to related enzymes and substrates. Here, the development of a potent and highly selective two-step activity-based probe for cathepsin S and the application in multicolor bio-orthogonal correlative light-electron microscopy is presented. LHVS, which has been reported as a selective inhibitor of cathepsin S with nanomolar potency, formed the basis for our probe design. However, in competitive activity-based protein profiling experiments LHVS showed significant cross-reactivity toward Cat L. Introduction of an azide group in the P2 position expanded the selectivity window for cathepsin S, but rendered the probe undetectable, as demonstrated in bio-orthogonal competitive activity-based protein profiling. Incorporation of an additional azide handle for click chemistry on the solvent-exposed P1 position allowed for selective labeling of cathepsin S. This highlights the influence of click handle positioning on probe efficacy. This probe was utilized in multicolor bio-orthogonal confocal and correlative light-electron microscopy to investigate the localization of cathepsin S activity at an ultrastructural level in bone marrow-derived dendritic cells. The tools developed in this study will aid the characterization of the variety of functions of cathepsin S throughout biology.

## Introduction

Cathepsin S (Cat S) is a lysosomal cysteine endopeptidase from the papain-superfamily and is an important mediator of adaptive immunity (Jakoš et al., 2019). Cat S is mainly expressed in immune cells such as B cells, macrophages and dendritic cells (DCs) and to a lesser extent in some epithelial cells (Gupta et al., 2008). Unlike most cysteine cathepsins, Cat S shows activity over a broad pH range which aids in extracellular activity (Gupta et al., 2008). Cat S is responsible for extracellular matrix (ECM) breakdown, e.g., elastin, fibronectin, collagen I, II and IV, and cleavage of cell adhesion molecules, such as JAM-B, ALCAM and L1CAM among many others (Fonović and Turk, 2014; Sevenich et al., 2014; Sobotič et al., 2015). This assists immune cell motility and function, however Cat S overexpression by tumor and tumor-associated cells, in turn facilitates tumor growth, invasion and metastasis that can even surpass the blood brain barrier (Wang et al., 2006; Sevenich et al., 2014). Inhibition of these processes by small molecule inhibitors reduces cancer malignancy and improves survival in mouse models (Sevenich et al., 2014; Olson and Joyce, 2015; McDowell et al., 2020).

Besides taking part in immune cell motility and the processing of antigenic material (Driessen et al., 1999; Shen et al., 2004), one of the unique roles of Cat S in immunology is the processing of the invariant chain (Ii) during the maturation of major histocompatibility complex II (MHC II), a central step in class II antigen presentation (Driessen et al., 1999; Bania et al., 2003). This increases DC motility in a myosin II-dependent manner which allows DCs to migrate toward the lymph node (Faure-André et al., 2008). During this process, Cat S specifically mediates the cleavage of a 10-kDa Ii fragment (Ii p10), which removes the endoplasmic reticulum retention signal (Theron et al., 2017). Cat S is essential for this process and specific inhibition leads to accumulation of MHC II-li p10 complexes in DCs and B cells (Theron et al., 2017). The li p10 cleavage leavesmulticolor a heterogeneous set of class II-associated invariant chain peptides (CLIPs) at the peptide binding groove of MHC II until exchanged with high affinity antigenic peptides by the action of human leukocyte antigen DM (HLA-DM). Together, this enables the presentation of extracellular derived antigens to CD4^+^ T helper cells. This process is regulated by TLR activation and cytokine signaling, where proinflammatory signals, such as lipopolysaccharides, interleukin 1β, tumor necrosis factor *α* and interferons type I and II, promote Cat S activity resulting in increased MHC II dimerization and CD4^+^ T cell priming. On the contrary, anti-inflammatory signaling by IL10 counteracts this process (Fiebiger et al., 2001; Reich et al., 2007; Jakoš et al., 2019). The role of Cat S in T cell activation has also linked Cat S to the mechanism of neuropathic pain (Eckert et al., 2020), and Cat S cleavage of PAR2 causes TRPV4-dependent inflammatory pain (Zhao et al., 2014). Lastly, Cat S is an promising therapeutic target for the treatment of rheumatoid arthritis, psoriasis, asthma, and potentially cystic fibrosis and COPD (with several molecules at diverse stages of development) (Wilkinson et al., 2015; Brown et al., 2020).

To gain better understanding of the multifaceted biology of Cat S several research groups have developed imaging tools to detect Cat S activity. Since cathepsin activity is highly regulated, dependent on localization, proteolytic activation and endogenous inhibitors, standard biochemical techniques as genetic ablation, the assessment of RNA or protein levels, fail to outline when and where these proteases are active. An excellent method to detect proteolytic activity is the use of activity-based probes (ABPs). ABPs enable the enrichment or visualization of protease activity in a spatiotemporal fashion *in vitro* up to whole organisms (Sanman and Bogyo, 2014). An ABP typically consists of three parts, the most distinctive being the electrophilic trap, which is designed to react with the active-site nucleophile, covalently linking the probe to the enzyme. This ‘warhead’ is tethered to a recognition or linker element which can be designed to dictate target specificity of the ABP. Thirdly, a reporter tag is attached to facilitate detection. This tag can be a fluorophore, an affinity tag or a bio-orthogonal functional group, such as an alkyne or an azide. The latter tags are attractive because of their small size and flexibility in terms of conjugation of a variety of reporter tags in a second step after proteome labeling. Such probes are hence dubbed two-step ABPs.

Using DCG-0N (Greenbaum et al., 2000), a biotinylated ABP based on the natural product epoxysuccinyl E64, Reich et al. showed that endocytic uptake via macropinocytosis by human monocyte-derived dendritic cells (moDCs) routed peptide-size material selectively to active Cat S (Reich et al., 2007). DCG-0N in complex with streptavidin however, resulted in less strict targeting of Cat S and additionally labeled Cat B and X in moDCs. Lastly, conjugation of DCG-0N to a cell penetrating peptide abolished the labeling selectivity completely. This suggests differential compartmentalization of cathepsin activity, which could be regulated by phago/endosomal routing and maturation, directed by factors like specific receptor-mediated uptake or the size and molecular nature of the engulfed material. Bender and co-workers developed a selective Cat S quenched ABP (qABP) for live cell imaging and non-invasive *in vivo* applications (Oresic Bender et al., 2015). This helped define Cat S activity in an orthotopic mouse model of breast cancer and was able to selectively label Cat S in various tissues. Contrary to bone marrow-derived macrophages, dual color cathepsin activity labeling experiments in naïve mouse bone marrow-derived dendritic cells (BMDCs) revealed vesicles in which exclusively Cat S activity could be detected, without labeling of Cat X, B and L activity. The exact structure and function of these Cat S vesicles remain elusive. Van Elsland and co-workers recently reported on the combination of bio-orthogonal labeling with high-resolution correlative light-electron microscopy (CLEM) to detect activity of cysteine cathepsins in mouse BMDCs with the pan-reactive two-step ABP DCG-04-azide (Elsland et al., 2015). As expected, cathepsin labeling was fully contained within membrane-limited structures and partly overlapped with LAMP1 lysosomal staining. Here we set out to develop a highly selective two-step ABP to pinpoint the activity of Cat S using multicolor bio-orthogonal confocal microscopy and CLEM.

## Materials and Methods

### ABP Synthesis

**SCHEME 1:**
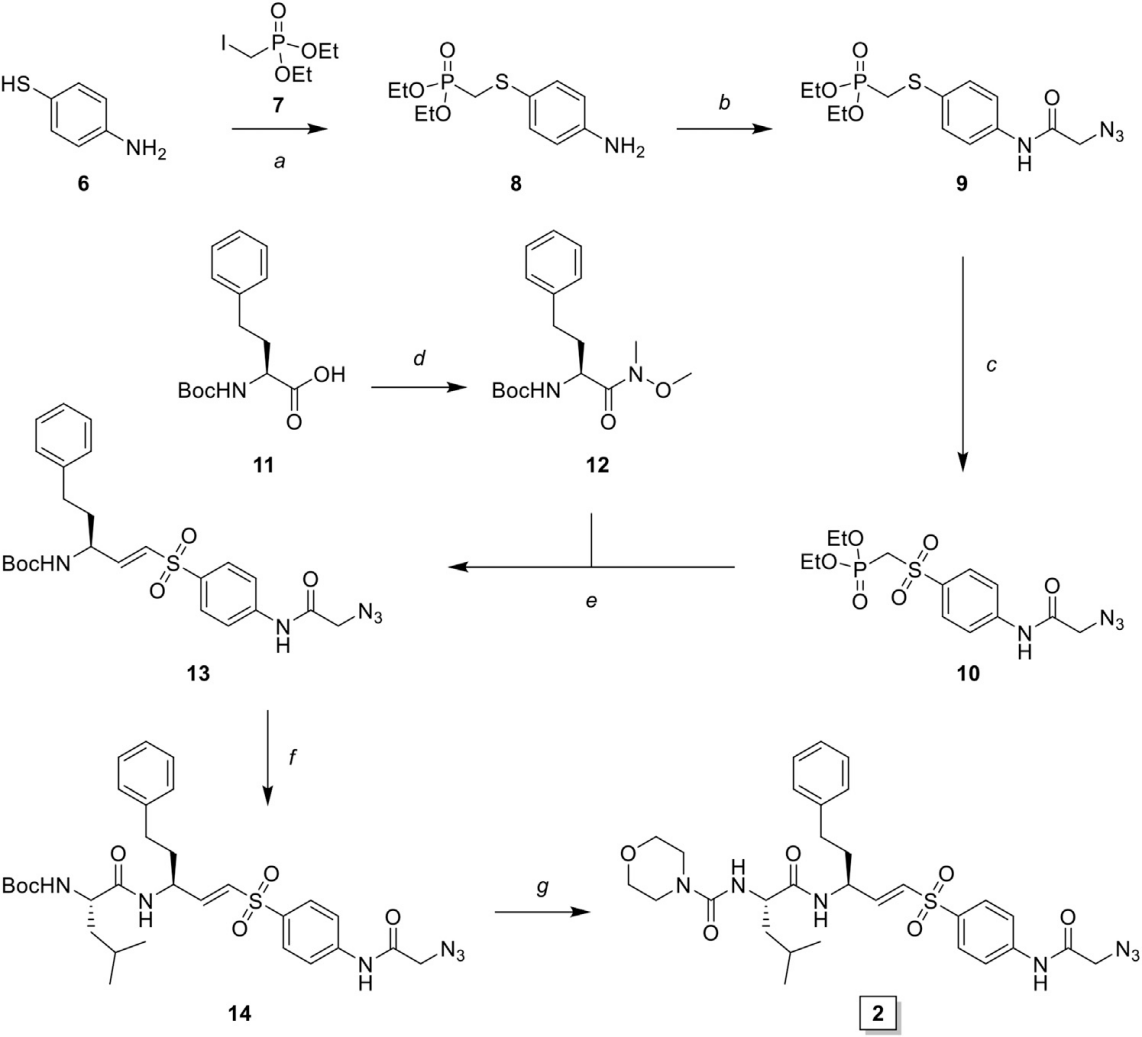
Synthesis of LHVS derivative 2 (FD054). *Reagents and conditions*: **(A)** NaH, THF, 0°C, 15 min, then diethyl (iodomethyl)phosphonate, THF, 0°C to r. t., 72 h, quant.; **(B)** *i*. bromoacetyl bromide, DiPEA, DCM, 0°C, 1 h *ii*. NaN_3_, DMF, r. t., 4 h, quant.; **(C)** AcOOH, DCM, 0°C to r. t., 4 h, 93%; **(D)** DMHA·HCl, HBTU, DiPEA, DMF, r. t., 1 h, quant.; **(E)** LiAlH_4_, THF, 0°C, 15 min, 94%; **(F)** phosphonate **3**, NaH, THF, -10°C, 15 min, then aldehyde **12**, THF, -10°C to r. t., 16 h, 42%; **(G)** *i*. TFA/DCM, r. t., 30 min, *ii*. Boc-l-Leu-OSu, DiPEA, DMF, r. t., 4 h, 95%; **(H)** *i*. morpholine, DiPEA, triphosgene, THF, 0°C, *ii*. carbamate 7, TFA/DCM, r. t., 30 min, *iii*. crude carbonyl chloride, DiPEA, DMAP (cat.), THF, r. t., 16 h, 32%.

**SCHEME 2:**
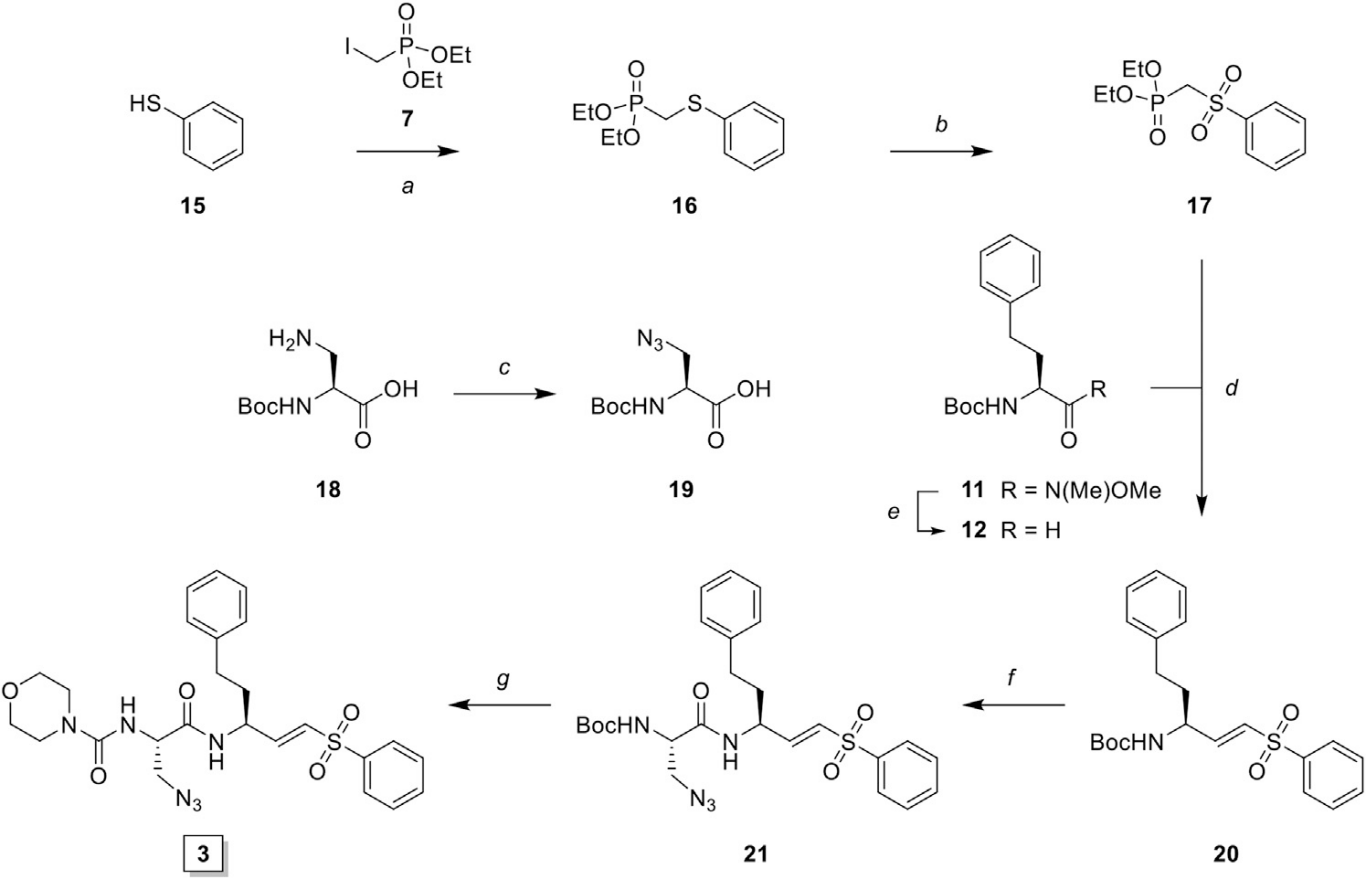
Synthesis of LHVS derivative **3** (FD062). *Reagents and conditions*: **(A)** NaH, THF, 0°C, 15 min, then diethyl (iodomethyl)phosphonate, THF, 0°C to r. t., 2 h, quant.; **(B)** AcOOH, DCM, 0°C to r. t., 4 h, 95%; **(C)** imidazole-1-sulfonyl azide, K_2_CO_3_, CuSO_4_·5H_2_O (cat.), MeOH, r. t., 16 h, 71%, **(D)** LiAlH_4_, THF, 0°C, 15 min, 89%; **(E)** phosphonate, NaH, THF, -10°C, 15 min, then crude aldehyde **12**, THF, -10°C to r. t., 3 h, 70%; **(F)** *i*. TFA/DCM, r. t., 30 min, *ii*. Boc-L-Aza-OH, HBTU, DiPEA, DMF, r. t., 16 h, quant.; **(G)** *i*. TFA/DCM, r. t., 30 min, *ii*. 4-morpholine carbonyl chloride, DiPEA, DMF, r. t., 16 h, 31%.

**SCHEME 3:**
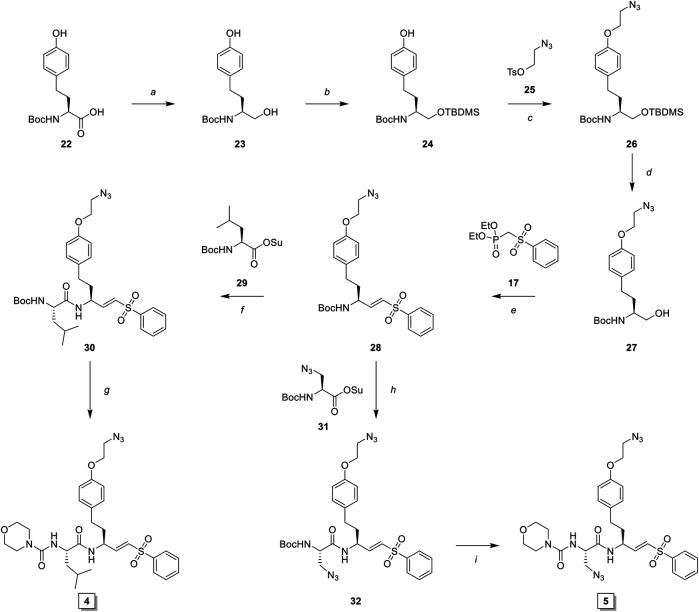
Synthesis of LHVS derivative **4** (FJD237) and **5** (FJD239). *Reagents and conditions*: **(A)** borane, THF, 0°C, 5 h, quant.; **(B)** *i*. TBDMSCl, imidazole, DMF, r. t., 30 min, *ii*. TBAF, THF, 0°C, 1 h, quant.; **(C)** NaH, DMF, -10°C to r. t., 1 h, 95%; **(D)** TBAF, THF 0°C, 15 min, 98%; **(E)** *i*. Oxalyl chloride, DMSO, Et_3_N, DCM, -78°C to r. t., 30 min, *ii*. NaH, THF, then crude aldehyde, -10°C to r. t., 30 min, 90%; **(F)** *i*. TFA/DCM, 30 min, r. t., *ii*. Boc-L-Leu-OSu, DiPEA, DMF, 16 h, r. t., 93%; **(G)** *i*. TFA/DCM, 30 min r. t., *ii*. morpholine carbonyl chloride, DiPEA, DMF, 16 h, r. t., 55%; **(H)** *i*. TFA/DCM, 30 min, r. t., *ii*. Boc-L-Aza-OSu, DiPEA, DMF, 16 h, r. t., 77%; **(I)** *i*. TFA/DCM, 30 min, r. t., *ii*. morpholine carbonyl chloride, DiPEA, DMF, 16 h, r. t., 51%.

Compounds 2–5 were synthesized as depicted in Schemes 1–3. Detailed experimental procedures and analytical data can be found in the Supplemental Material.

### Cell Culture

RAW cells (264.7) were maintained in DMEM (Gibco®) containing high glucose, stable glutamine (GlutaMAX™), sodium pyruvate and phenol red, which was supplemented with 10% fetal calf serum (FCS, Bio-Greiner One) and antibiotics (100 units/ml penicillin, 100 μg/ml streptomycin, and 250 ng/ml amphotericin B (Gibco®)). The cells were cultured in a humidified 5% CO_2_-atmosphere at 37°C and the culture was passaged every 2–3 days. The cells were suspended via a cell-scraper, or by incubation with 10 mM EDTA for 5 min at 37°C, followed by centrifugation at 1,000 × *g* for 5 min at 4°C. The medium was refreshed and the cells were seeded to the appropriate confluence. Cells were harvested at 80–90% confluence.

Mouse bone marrow was isolated from the femur and tibia from 8–12 weeks old C57BL/6 mice. Bone marrow-derived dendritic cells (BMDCs) were obtained by differentiation under influence of recombinant mouse granulocyte macrophage colony stimulating factor (GM-CSF) (25 ng/ml) in RPMI medium containing HEPES, stable glutamine and phenol red, which was supplemented with fetal bovine calve serum (FCS) (10%), *ß*-mercaptoethanol (50 µM) and antibiotics (100 units/ml penicillin, 100 μg/ml streptomycin, and 250 ng/ml amphotericin B (Gibco®)). The cells were cultured in a humidified 5% CO_2_-atmosphere at 37°C and the medium was supplemented every three days with GM-CSF (25 ng/ml, final concentration). The floating population was harvested after 7 days.

### cABPP in Whole Cells

RAW macrophages (2·10^5^ cells in 100 µL conditioned medium) were incubated with the indicated concentration of inhibitor (200x in DMSO) for 1 h at 37°C, followed by labeling with 1 µM BMV109 (200x in DMSO) for 1 h at 37°C. The cells were centrifuged at 10,000 × *g* for 1 min at r.t., the supernatant was removed, and the cells were taken up in 9 µL hypotonic lysis buffer (50 mM PIPES pH 7.4, 10 mM KCl, 5 mM MgCl, 4 mM DTT, 2 mM EDTA, and 1% NP40). The lysate was incubated on ice for 5 min, followed by centrifugation at 21,130x *g* for 15 min at 4°C. The cleared lysate was diluted with 3 µL Laemmli’s 4x sample buffer (40% glycerol, Tris/HCl (0.2 M, pH 6.8), 8% SDS, 10% BME, and 0.04% bromophenol blue) and the mixture was denatured over 5 min at 95°C. The samples were spun down and separated by SDS PAGE (15%, 15 min at 80 V, 1.5–2 h at 120 V). The gel was analyzed by in-gel fluorescence scanning on a Typhoon Trio flat-bed laser scanner (GE Healthcare) and equal protein loading was confirmed by staining with Coomassie® Brilliant Blue R-250 (Schmidt GmbH).

### Copper-Click cABPP in Whole Cells

Intact RAWs or BMDCs (some 2·10^5^ cells in 100 µL conditioned culture medium) were incubated with the indicated concentration of inhibitor (200x in DMSO) for 1 h at 37°C, prior to labeling with 1 µM BMV109 (200x in DMSO) for 1 h at 37°C. The cells were centrifuged (10,000x g for 1 min at r. t.), the supernatant was removed, and the cells were taken up in 10 µL lysis buffer (50 mM HEPES pH 7.3, 10 mM KCl, 5 mM MgCl_2_ and 1% NP40). The lysate was incubated on ice for 15 min followed by centrifugation at 21,130 × g for 15 min at 4°C. The conditioned lysate was incubated with 10 µL click-cocktail (50 mM HEPES pH 7.3, 1 mM CuSO_4_, 10 mM sodium ascorbate, 1 mM THPTA ligand, 10 mM amino-guanidine, 5 μM AF488 alkyne (Click Chemistry Tools)) for 1 h at 20°C. The protein was pelleted by addition of 80 µL cold acetone followed by incubation at −20°C for 30 min. The lysate was centrifuged (21,130 × g for 15 min at 4°C), the supernatant was removed and the samples were taken up in 15 µL Laemmli’s 1x sample buffer (10% glycerol, 50 mM Tris/HCl pH 6.8, 2% SDS, 2.5% BME, and 0.01% bromophenol blue), denatured for 5 min at 95°C and separated by SDS PAGE (15%). The gel was analyzed by in-gel fluorescence scanning on a Typhoon Trio (GE Healthcare) flat-bed laser scanner and equal protein loading was confirmed by staining with Coomassie® Brilliant Blue R-250 (Schmidt GmbH).

cABPP-labelling intensities were quantified using ImageJ software (Schneider et al., 2012). Data was transferred to Microsoft Excel, corrected for background fluorescence, and scaled to the positive control (DMSO, BMV109) as 100%-activity reference point. The mean, standard deviation (SD), and standard error of the mean (SEM) were calculated and normalized to the corrected positive control. The data was transferred to Graphpad Prism 6.0 and IC50-values were calculated using non-linear regression.

### Confocal Microscopy

BMDCs were seeded (2·10^5^) on an 8-well chamber slide (Ibidi) and left to grow for 2 h. Next *E. coli* B834-cells harboring Hpg were added to the cells in a 50:1 ratio. After 1 h, the cells were washed three times with PBS, and fresh medium was added. The cells were incubated with 100 nM probe 5 (200x in DMSO) for 1 h after which the cells were washed three times with PBS, then incubated with 1 µM BMV109 (200x in DMSO) for 1 h and washed again. The cells were fixed in 4% PFA for 2 h and until further analysis cells were kept in 0.5% PFA in PBS at 4°C. When all slides were collected, fixed cells were washed with PBS and PBS containing 20 mM glycine (PBS/glycine). Membrane permeabilization was achieved by incubating with 0.1% Triton-X100 for 20 min, followed by a PBS wash. Dual copper-catalyzed Huisgen cycloaddition (ccHc) reaction was performed sequentially with intermediate PBS washes as described previously (Bakkum et al., 2020), with click cocktail 1 ((0.1 M HEPES, pH 7.3, 1 mM CuSO_4_, 10 mM sodium ascorbate, 1 mM THPTA ligand, 10 mM amino-guanidine, 5 µM AFDye555-azide (Click Chemistry Tools)) for 30 min to label Hpg-*E. coli*, followed by click cocktail 2 ((0.1 M HEPES, pH 7.3, 1 mM CuSO_4_, 10 mM sodium ascorbate, 1 mM THPTA ligand, 10 mM amino-guanidine, 5 µM AF488-alkyne (Thermo Fisher)) for 30 min to label Cat S. Cells were washed with PBS and blocked with PBS containing 1% BSA for 30 min to assist in removing non-specifically bound fluorophores. After two more PBS washes, the nuclei were counterstained with 2 μg/ml Hoechst 33342 for 5 min and washed once more with PBS, prior to imaging in glycerol/DABCO solution on an Andor Dragonfly 505 Spinning Disk Confocal (Oxford Instruments), containing an 8-line integrated laser engine. Images were acquired with the Zyla 2048 × 2048 sCMOS camera and 2 × 2 camera binning controlled with the integrated Fusion software. Z-series optical sections were collected with a system-optimized step-size of 0.13 microns and were deconvolved using the integrated ClearView-GPU™ deconvolution software. Z-series are displayed as maximum z-projections, and gamma, brightness and contrast were carefully adjusted (identically for compared image sets) using FIJI (Schindelin et al., 2012).

### B-CLEM–Sample Preparation

The sample preparation for bio-orthogonal correlative light-electron microscopy (B-CLEM) and bio-orthogonal labeling was performed as previously described (Bakkum et al., 2020). Briefly, following infection and post-infection incubation with the cathepsin probes, BMDCs were washed three times with conditioned medium and once with PBS. The cells were fixed with TEM-optimized fixation solution (2% EM-grade paraformaldehyde, 0.2% EM-grade glutaraldehyde in 0.1 M phosphate buffer pH 7.2; Electron Microscopy Sciences) for 2 h at r. t., washed with PBS and stored in storage buffer (0.5% EM-grade paraformaldehyde in 0.1 M phosphate buffer pH 7.2) at 4°C until all time points were collected. Fixed cells were rinsed with PBS and PBS/glycine, harvested in warm 1% gelatin (type A, bloom 300; Sigma-Aldrich) in PBS via cell scraper and transferred to a 15 ml Falcon tube, prior to collection by centrifugation. The pellet was suspended in warm 1% gelatin in PBS (100 µL), transferred to a 1.5 ml Eppendorf tube. Warm 12% gelatin in PBS was added (1 ml) and the cells were pelleted by centrifugation (3 min at 800 × g). The pellets were jellified on ice and the sample pellet was cut off from the tube and cut in half with a surgical blade. Sample cubes of approx. 1 mm^2^ were prepared and rotated in a 2.3 M sucrose solution for 18 h to allow for sucrose infiltration. This was followed by plunge-freezing the cubes on metal support pins.

### B-CLEM–Cryo-Sectioning and on Section staining

Ultrathin (75 nm) cryo-sections were prepared according to the Tokuyasu technique (Tokuyasu, 1973), using a Leica UC7 Ultramicrotome equipped with Leica EM FC7 Cryochamber and Leica EM Crion ionizer/charger, Diatome Micromanipulator, Diatome Trim 20° knife and a Diatome Cryo Immuno 35° knife.. After collection, the sections were thawed on a droplet of pickup fluid (1.15 M sucrose, 1% methylcellulose) and transferred to a Formvar/carbon-coated TEM grid (titanium, 100 square mesh, 3.05 mm, center-marked; Agar Scientific), pre-coated with blue 0.2 µm FluoSpheres (Thermo Fisher) as fiducial markers. These on-grid cryo-sections were incubated on droplets of 2% gelatin in PBS for 30 min at 37°C, followed by washing 5 times on PBS/glycine. The subsequent dual ccHc reactions were performed as described above, followed by three washes with PBS. The sections were then incubated on droplets of 1% BSA in PBS for 3 × 10 min at r. t., to assist the removal of non-specifically bound fluorophores. Sections were additionally blocked on 0.1% BSA-c (Aurion) in PBS before incubating 1 h on anti-MHC II (M5/114, 1:300, BioXCell) or anti-LAMP-1 (1D4B, 1:300, eBioscience) in 0.1% BSA-c, followed by 20 min on a rabbit-anti-rat (1:50, Abcam) bridging antibody, and 20 min on protein-A conjugated to 15 nm gold (1:50; Aurion), with intermediate washes on 0.1% BSA-c. Sections were subsequently washed three times on PBS and antibody complexes immobilized by incubating 5 min on 1% glutaraldehyde, followed by additional PBS washes. Nuclear staining of the host cells was achieved by incubating the sections on a droplet of 0.2 μg/ml DAPI (Thermo Fisher) for 5 min.

### B-CLEM–Confocal Microscopy, TEM Imaging and correlation

After several washes with PBS, the fluorescently labelled sections on TEM grids were mounted in water containing 30% glycerol between a microscopy slide and a coverslip, and imaged by confocal microscopy as described above. The TEM grids containing the sections were recovered, rinsed in distilled water, and incubated for 5 min on droplets of uranyl acetate/methylcellulose. These sections were then imaged on a FEI Tecnai 12 BioTwin TEM System (FEI Technologies) at 120 kV acceleration voltage. Correlation of FM and TEM images was performed in Adobe Photoshop CC 2020 (Adobe Systems) as described previously (Bakkum et al., 2020).

## Results

### Synthesis of First Generation LHVS-Derived Two-step ABPs

**FIGURE 1:**
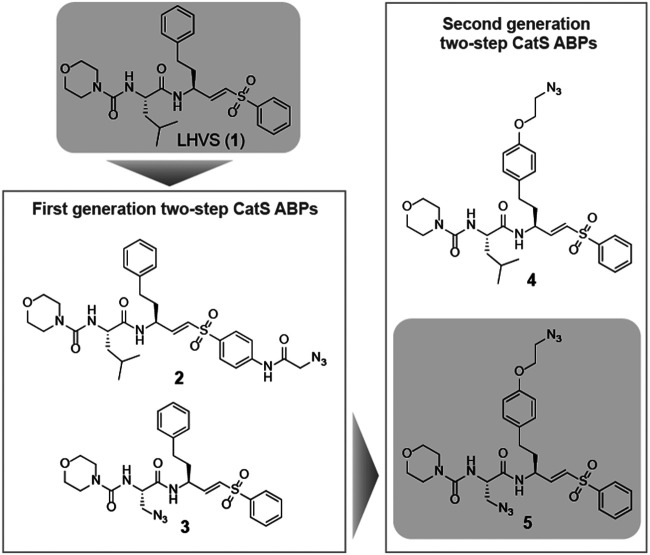
Structures of the reported Cat S selective inhibitor LHVS (1) and the first and second generation LHVS-derived two-step ABPs.

Morpholine leucine homophenyl vinyl sulfone (LHVS) (**1**, Figure 1) was selected as a starting point for the development of a two-step ABP. LHVS has been reported as a highly selective, nanomolar potent inhibitor of Cat S with near diffusion-limited kinetics (Palmer et al., 1995). Because vinylsulfones are effectively inert in the absence of an enzyme’s catalytic machinery, they represent an excellent class of electrophilic traps to facilitate selective labeling (Palmer et al., 1995). In a first attempt to synthesize LHVS-derived two-step ABPs two target molecules were designed, in which an azide handle was introduced to facilitate click chemistry (Kolb et al., 2001), either by extending the molecule on the phenyl vinylsulfone or in the P2 position, resulting in compounds **2** and **3**, respectively (Figure 1).

LHVS derivative **2** was synthesized using a modified procedure of the vinylsulfone synthesis described by Van der Linden and colleagues (van der Linden et al., 2015). In short, 4-aminothiophenol **6** was substituted with diethyl (iodomethyl)phosphonate **7** yielding thioether **8** (Scheme 1). Condensation with bromoacetyl bromide followed by substitution with sodium azide yielded azide **9** and subsequent oxidation with peracetic acid yielded phosphonate **10**. Boc-l-homophenylalanine was converted into its corresponding aldehyde **12** by reduction of the Weinreb amide with lithium aluminum hydride and subsequent Horner-Wadsworth-Emmons (HWE) olefination with phosphonate **10** furnished vinylsulfone **13**. Boc-deprotection followed by condensation with Boc-l-Leu-OSu and subsequent Boc-deprotection and coupling with morpholine carbonyl chloride yielded LHVS derivative **2** (FD054). Similarly, thiophenol **15** was substituted with phosphonate **7** followed by oxidation with peracetic acid to form **17** (Scheme 2). HWE olefination with aldehyde **11** yielded vinyl sulfone **20** and sequential Boc-deprotections, and subsequent coupling with Boc-l-Aza-OSu **19** and morpholine carbonyl chloride, respectively, afforded compound **3** (FD062).

### *In vitro* Performance of the First Generation LHVS-Derived Two-step ABPs

**FIGURE 2:**
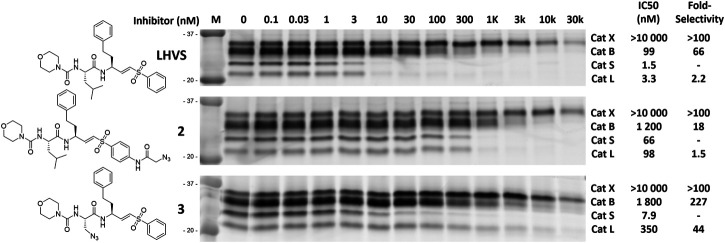
Competitive labeling of LHVS derivatives. Live RAW macrophages were incubated with the indicated concentration of inhibitor for 1 h after which the residual cathepsin activity was labeled with BMV109. The cells were lyzed, separated by SDS PAGE and the labeling intensity was quantified. IC50s and selectivity for Cat S were calculated and are presented in the adjacent table. Experiments were performed in duplicate.

**FIGURE 3:**
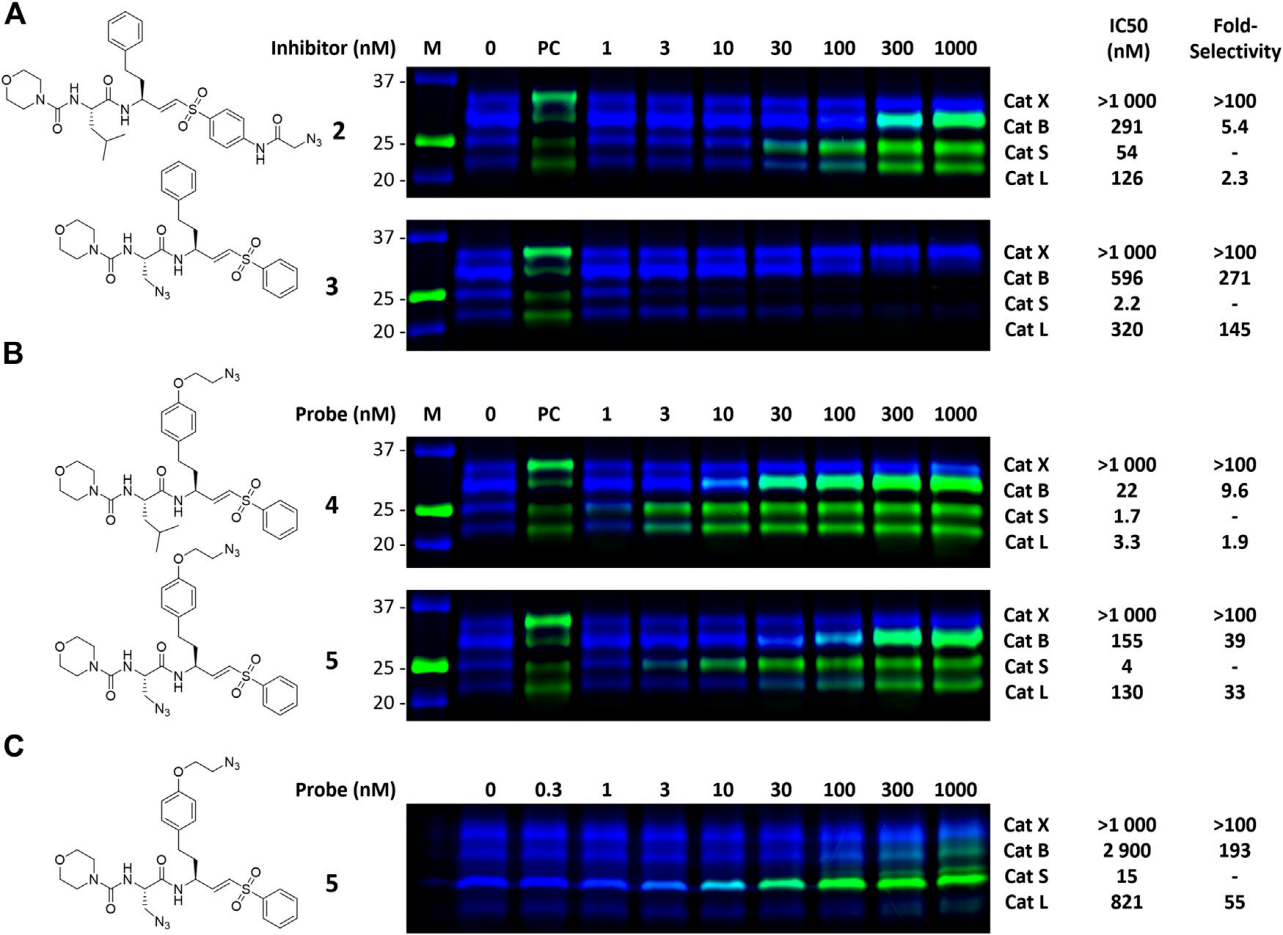
Dual color competitive labeling **(A) (B)** Whole RAW cells were incubated with indicated concentration of inhibitor/probe for 1 h and residual cathepsin activity was labeled with BMV109 (blue) over 1 h. Cells were lyzed and the proteomes were labeled with AF488-alkyne (green) using copper-catalyzed click chemistry **(C)** Mouse bone marrow-derived dendritic cells were incubated with the indicated concentration of inhibitor followed by labeling with BMV109 (blue) and click chemistry with AF488-alkyne (green). IC50s were determined by analysis of the competitive labeling by BMV109 and are given in nanomolar. The fold-selectivity for Cat S is calculated from the apparent IC50s. Experiments were performed in duplicate. FJD164 (Supplementary Scheme S1) was used as positive control (PC) for click labeling.

To investigate the inhibition efficiency of the synthesized LHVS derivatives a competitive activity-based protein profiling (cABPP) assay was performed in 264.7 RAW cells (a mouse macrophage cell line). Live RAW cells were incubated with increasing concentrations of LHVS (**1**), **2** and **3**, after which residual cathepsin activity was labeled with pan-reactive qABP BMV109 (Verdoes et al., 2013). Cells were lyzed and the proteome was separated by SDS-PAGE. Next, in-gel fluorescence scanning and quantification of the decrease in labeling intensity yielded the apparent half-maximal inhibitory concentrations (IC50s) (Figure 2). Unexpectedly, this revealed that LHVS has significant off-target activity against Cat L, in addition to its nanomolar inhibition of Cat S. Apparently, the reported inhibitory constants (K_i_) and potencies (K_inact_/K_i_) (Palmer et al., 1995) fail to reflect the eventual inhibitory potency and selectivity in a whole cell setting. Introduction of an azide tag on the phenyl vinylsulfone in probe two resulted in some 40-fold decrease in potency for Cat S, while cross-reactivity with Cat L was maintained and the selectivity window with Cat B was decreased. Coincidently, cABPP revealed that the replacement of the P2 leucine of LHVS with azidoalanine in compound **3** increased the selectivity window, leading to some 40-fold selectivity for Cat S in respect to Cat L, albeit at the cost of a slight (some 4-fold) reduction in potency toward Cat S. Unfortunately, subsequent two-step labeling experiments showed that compound **3** in complex with Cat S was unable to be labeled with Alexa Fluor 488 (AF488) alkyne (Figure 3A). That while probe **2** showed a concentration-dependent increase in AF488 fluorescence intensity, correlating with the decrease in Cy5 fluorescence due to the competition with BMV109 labeling (Figure 3A). Even upon harsh denaturing conditions Cat S modified with **3** could not be labeled with AF488 alkyne (results not shown).

**FIGURE 4:**
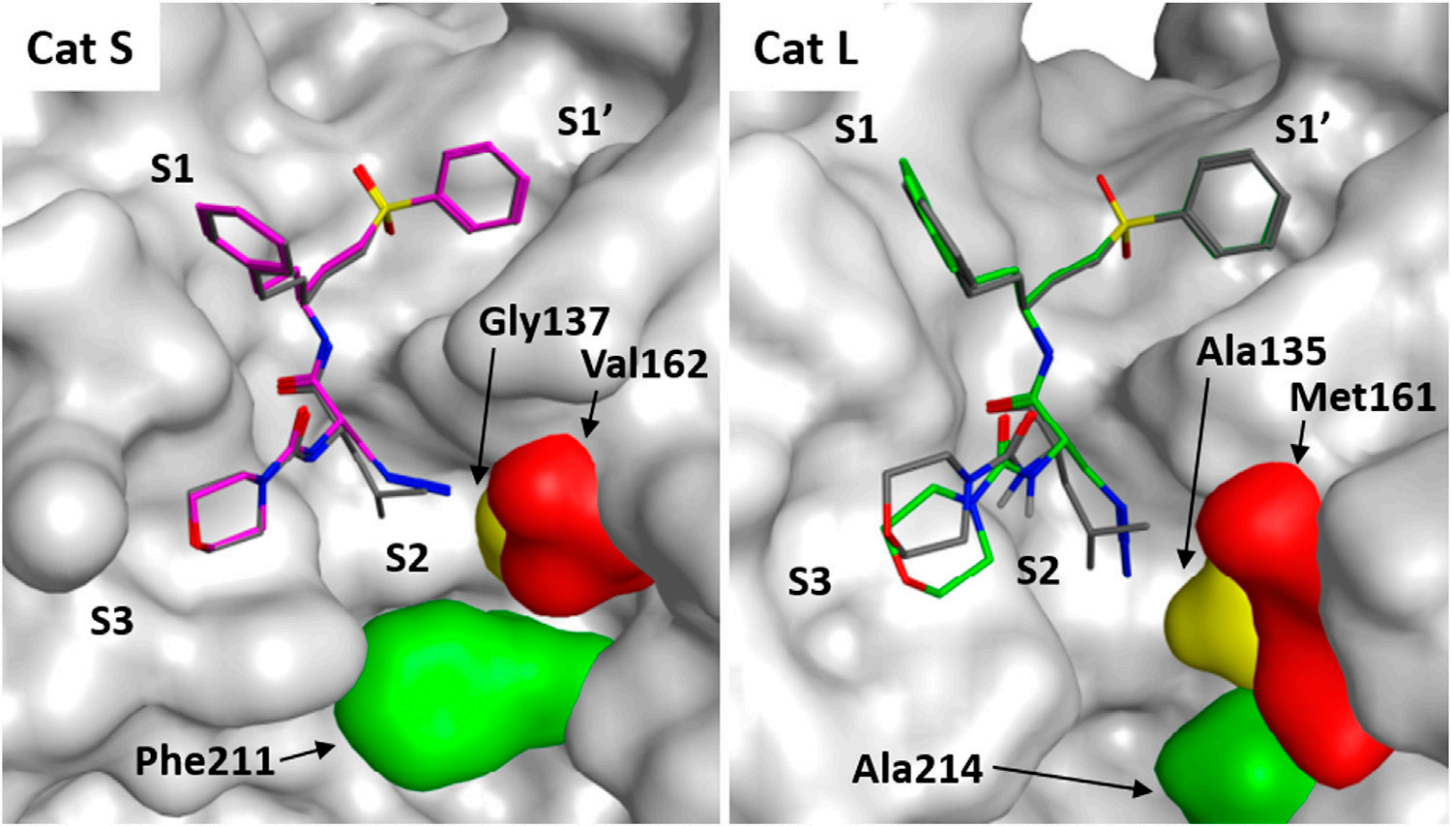
Molecular docking of LHVS analogues in cathepsin S and L. Molecular docking of LHVS (gray) and **3** (purple) in cathepsin S reveals a near identical binding mode for both inhibitors. Whereas docking overlay of LHVS (gray) and **3** (green) in cathepsin L reveals a significant clash between the azide group and Ala135 in cathepsin L distorting the binding mode. Molecular docking and image preparation was performed with MOE molecular dynamics software.

To gain further insight in the difference between LHVS and **3** with respect to binding interactions with Cat S and L, a docking study was performed based on the reported crystal structure of LHVS in complex with Cat S (Pauly et al., 2003). From this crystal structure three residues can be defined in the S2 pocket that are unique to Cat S, which might confer specificity in respect to Cat L (Supplemental Figure S1 and Figure 4). The entrance of the S2 site is governed by a *ß*-branched valine 162, which makes the entry of the S2 pocket narrower in Cat S in comparison with Cat L where methionine 161 is found in this position (Figure 4). This open S2 structure of Cat L likely facilitates the cross-reactivity of the P2 leucine toward Cat L. Secondly, the S2 subsite in Cat S is more plastic, governed by phenylalanine 211 which can fold away to accept bulky substituents (Pauly et al., 2003). In Cat L a completely open structure is provided by an alanine 214 residue, which confers limited options for selectivity between the two enzymes. Lastly, the S2 pocket in Cat S is deeper where extra space is governed by glycine 137 in comparison with alanine 135 in Cat L. Specifically this alanine distorts the docking of the P2 azide in Cat L (Figure 4). This subsequently disrupts the binding of the S3 morpholine in the P3 pocket. We hypothesize that the slight reduction in potency toward Cat S might follow from the loss of Van Der Waals interactions between the hydrophobic S2 pocket and the P2 azidoalanine in compound **3** relative to the leucine in LHVS. Together, this could help explain the improved selectivity for Cat S of compound **3** as observed in the cABPP experiment.

### Synthesis of the Second Generation LHVS-Derived Two-step ABPs

The observed Cat S selectivity of compound **3** encouraged the design of a second generation LHVS-derived two-step ABPs in which a solvent-exposed azide was introduced to enable efficient bio-orthogonal labeling. Extension of the P1 homophenylalanine of LHVS and inhibitor **3** with an azidoethyl ether results in target compounds **4** and **5**, respectively (Figure 1). Synthesis commenced from Boc-l-homotyrosine **22** to facilitate the incorporation of an azide group on the phenyl ring (Scheme 3). In order to mitigate reduction of the azide group during preparation of the required aldehyde, an alternative to the Weinreb-amide route was designed based on Swern oxidation of the corresponding alcohol.

Boc-l-homotyrosine was reduced with borane in THF and global protection with tert-butyldimethylsilyl chloride followed by regio-selective deprotection of the phenolic silyl ether with tetra-n-butylammonium fluoride (TBAF) furnished phenol **24**. 2-Bromoethanol was reacted with sodium azide and subsequently with 4-toluenesulfonyl chloride to form 2-azidoethyl tosylate **25**, which was reacted with phenol **24** to yield azide **26**. Next, deprotection with TBAF gave alcohol **27**, which was oxidized to the aldehyde via Swern oxidation, followed by HWE olefination with phosphonate **17** to produce vinylsulfone **28**. Next, probe **4** (FJD237) was obtained by subsequent Boc-deprotection, coupling with Boc-l-leucine-OSu followed with Boc-removal and coupling with morpholine carbonyl chloride. Similarly, sequential Boc-deprotections and coupling with Boc-l-azidoalanine-OSu and morpholine carbonyl chloride, respectively, yielded probe **5** (FJD239).

### Dual Color cABPP With Second Generation LHVS-Derived Two-step ABPs

With the synthesized second generation probes in hand, a dual color two-step labeling cABPP experiment was performed in RAW macrophages (Figure 3B). After incubation with live cells and subsequent cell lysis, the labeled proteomes were subjected to copper-catalyzed click chemistry with AF488 alkyne to visualize the formed probe-cathepsin conjugates. Compared to LHVS, the introduction of the azidoethyl ether in probe **4** did not majorly effect the inhibitory potency. Robust two-step labeling of Cat B, S and L was detected in the AF488 channel. Cat X was unaffected at the concentrations used. Di-azido-probe **5** showed an inhibition profile similar to inhibitor **3**, with a good apparent selectivity window for Cat S, albeit slightly less potent and selective, possibly as a consequence of the introduction of the azidoethyl ether in P1. As seen for probe **4**, two-step labeling of probe **5** was robust, with a good window for selective Cat S labeling between 10 and 100 nM. Next, probe **5** was tested in naïve mouse BMDCs to investigate the selectivity for Cat S in this cell type (Figure 3C). BMDCs have an increased relative expression of Cat S, which in turn increased the selectivity of the labeling to over 50-fold. Saturated labeling can be observed at a concentration of 100 nM without any off-target labeling in BMDCs. This shows that probe **5** allows selective saturation and labeling of Cat S activity.

### Multicolor Bio-Orthogonal Confocal Microscopy and Correlative Light-Electron Microscopy

**FIGURE 5:**
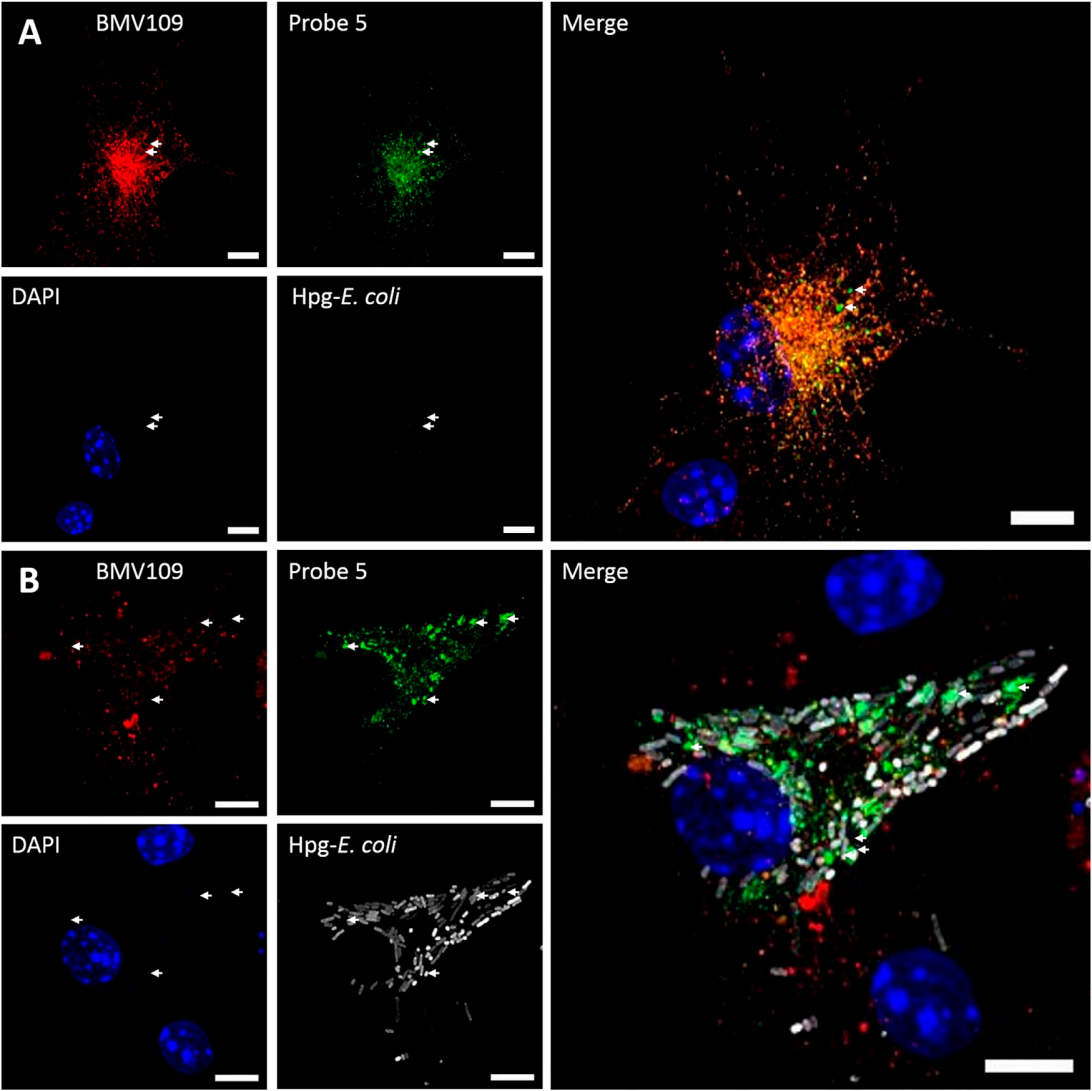
Dual click labeling in confocal microscopy **(A)** Naïve BMDCs (at time point 0) were treated subsequently with probe **5** (green) for 1 h and BMV109 (red) for 1 h, and probe **5** was visualized through click chemistry **(B)** BMDCs were treated with Hpg-labelled *E. coli* (white) for 3 h followed by probe **5** (green) and BMV109 (red) labeling ensued by tandem click chemistry. Specialized Cat S vesicles are annotated with white arrows. Scale bar represents 5 µm.

To address whether probe **5** would be applicable to image cellular localization of Cat S activity via bio-orthogonal conjugation of a fluorescent dye, we performed a set of pilot confocal microscopy experiments with mouse BMDCs which were treated with probe five and the broad-spectrum probe BMV109 sequentially (Figure 5A). Furthermore, BMDCs were given homopropargylglycine labeled *E. coli* (Hpg-*E. coli*) for 3 h prior to labeling with the aforementioned ABP-cocktail (Figure 5B). This would allow us to assess whether probe **5** could also be imaged in a ‘dual-click’ approach with the potential to determine the distribution of Cat S activity in response to, and relative to this bacterial stimulus (Bakkum et al., 2020). We noted a high variance in cathepsin activity between BMDCs, which might follow from the fact that GM-CSF-generated BMDCs form a highly heterogeneous population of monocytic cells (Helft et al., 2015). We also noted that, intact bacteria can still be observed at 3 h post infection, and within this timeframe no specific redistribution of either probe **5** or BMV109 to whole *E. coli*-containing vesicles could be observed. Furthermore, we observed that most vesicles labeled positive for both Cat S activity and the pan-reactive probe BMV109. Some vesicles can be observed that exclusively contain Cat S activity without detectable activity of Cat X, B and L (Figure 5, white arrows), which is in line with the observation of Bender and co-workers (Oresic Bender et al., 2015). Nonetheless, the exact function and nature of these vesicles remain elusive.

**FIGURE 6:**
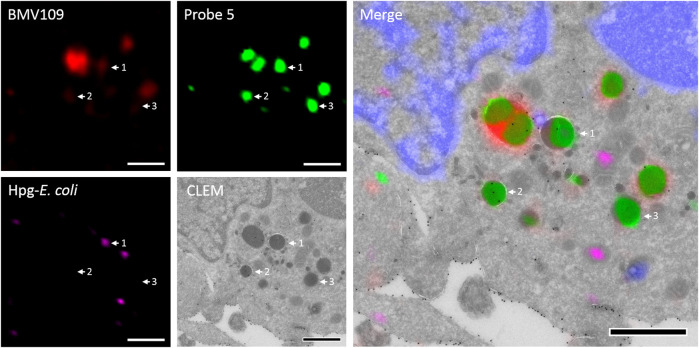
Bio-orthogonal correlative light-electron microscopy. BMDCs were treated with Hpg-*E. coli* (purple) followed by treatment with probe **5** (green) and BMV109 (red). Cryosectioning was followed by on-section tandem click reactions and MHC II was visualized with 15 nm immunogold labeling. The nuclei were stained with DAPI (blue) and the confocal and TEM pictures were correlated. Scale bar represents 1 µm.

To assess the feasibility of providing ultrastructural context to Cat S labeling, a pilot bio-orthogonal CLEM (B-CLEM) experiment was performed (Elsland et al., 2015). In this experiment, 75 nm sections of the BMDCs exposed to Hpg-E. Coli were taken after incubation with **5** and BMV109, followed by on-section sequential click reactions to fluorescently label bacterial products and probe **5** in complex with Cat S. Furthermore, MHC II was labeled with 15 nm gold particles to be imaged in negative stain transmission electron microscopy. After correlation of the two images, the fluorescence signals could be assessed in their ultrastructural context (Figure 6). Although no biological conclusions can be drawn from this pilot feasibility study, it does illustrate the potential of four color, dual bio-orthogonal fluorescence imaging in combination with CLEM and immunogold labeling. In the example shown, apparent colocalization between Cat S activity, bacterial digests and MHC II immunogold labeling seems to occur (Figure 6, arrow 1). Also vesicles costained for MHC II and Cat S with negligible BMV109 labeling are visible ((Figure 6, arrow 2), as well as vesicles seemingly containing solely Cat S activity (Figure 6, arrow 3). Together, this exemplifies the great applicability of probe five in fluorescence microscopy and state-of-the-art dual B-CLEM.

## Discussion

Cat S is an important mediator of adaptive immunity and an attractive pharmaceutical target in for example cancer and inflammatory pain. To pinpoint Cat S activity relative to other cysteine proteases in microscopy experiments, there is a challenge of generating highly selective ABPs. In this work the development and application of a highly Cat S selective two-step ABP is presented. As a starting point for our probe design we chose the small molecule inhibitor LHVS, which has been reported and extensively applied as a selective inhibitor of Cat S. Of note, the selectivity of in particular irreversible inhibitors and probes is highly dependent on experimental conditions and the relative activity of potential off-targets. Based on the results in this study, the major difference in inhibitory constants (K_i_) and potencies (K_inact_/K_i_) as reported for recombinant Cat S and Cat L *in vitro* (120-fold selectivity) (Palmer et al., 1995), failed to adequately reflect the eventual inhibition selectivity in live RAW cells (2-fold selectivity) (Figure 2). To us, this emphasizes the vital importance of testing new potential selective compounds in a more complex setting such as live cells and validate the target engagement and on-target selectivity through for example cABPP with broad-spectrum ABPs. Coincidently, a first attempt to obtain an LHVS-derived two-step ABP by replacement of the P2 leucine with azidoalanine resulted in a highly selective Cat S inhibitor. However, positioning of the azide ligation handle in the S2 pocket renders compound **3** resistant to copper catalyzed azide alkyne cycloaddition, even after denaturation of the irreversibly inhibited enzymes. Hence, in order to accomplish selective labeling two azides had to be incorporated on the LHVS structure. This yielded the highly selective and potent two-step ABP **5** for Cat S.

A set of pilot feasibility experiment demonstrated that this probe could be readily applied to image the localization of Cat S activity in BMDCs, both by using conventional confocal microscopy as well as CLEM. These experiments highlight the applicability and potential of probe five in multi-color confocal microscopy and bio-orthogonal CLEM. Therefore, the probe developed in this study could be applied in delineating the role of active Cat S in the fields of cancer, autoimmune disorders, bacterial pathogenesis and inflammatory pain.

## Data Availability

The original contributions presented in the study are included in the article/Supplementary Material, further inquiries can be directed to the corresponding author.
