# Supplementary material for: Application of a Highly Selective Cathepsin S Two-step Activity-Based Probe in Multicolor Bio-Orthogonal Correlative Light-Electron Microscopy
Source: Front Chem. 2021 Feb 8;8:628433. doi: 10.3389/fchem.2020.628433 (PMC7903248; doi:10.3389/fchem.2020.628433)
Supplement: Supplementary file 1 [file presentation1.pptx]

## Slide 1
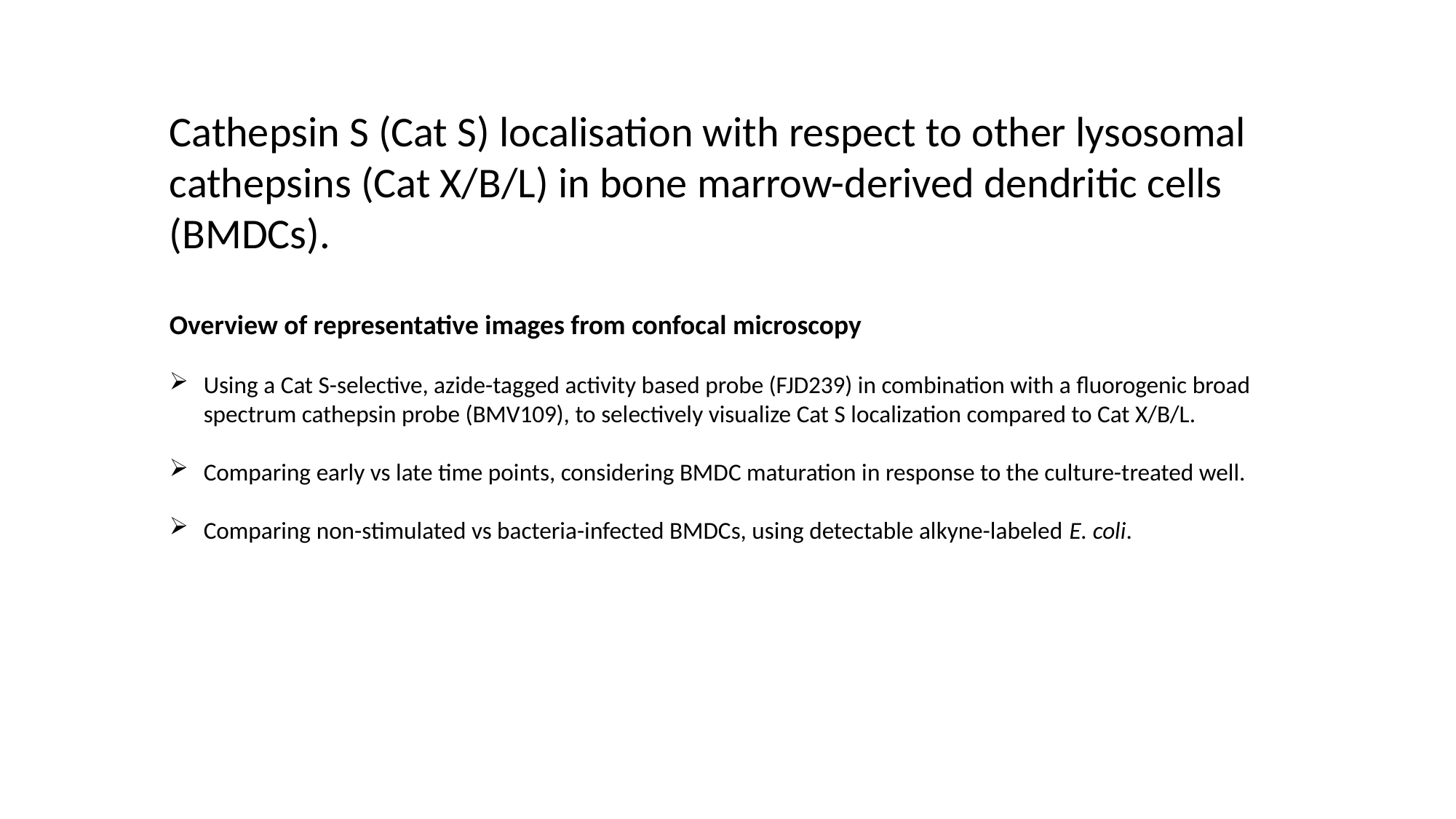

Cathepsin S (Cat S) localisation with respect to other lysosomal cathepsins (Cat X/B/L) in bone marrow-derived dendritic cells (BMDCs).
Overview of representative images from confocal microscopy
Using a Cat S-selective, azide-tagged activity based probe (FJD239) in combination with a fluorogenic broad spectrum cathepsin probe (BMV109), to selectively visualize Cat S localization compared to Cat X/B/L.
Comparing early vs late time points, considering BMDC maturation in response to the culture-treated well.
Comparing non-stimulated vs bacteria-infected BMDCs, using detectable alkyne-labeled E. coli.

## Slide 2
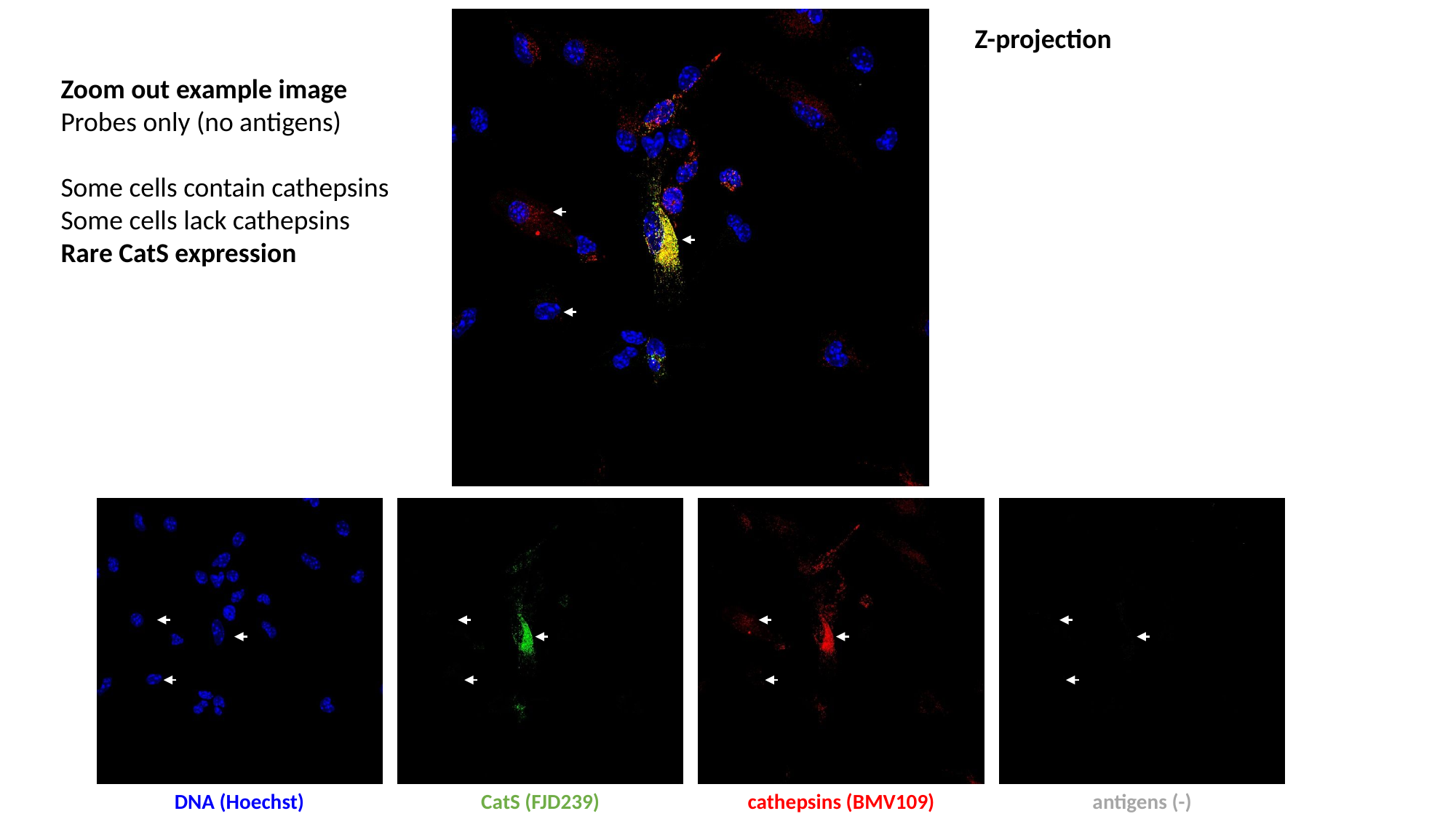

Z-projection
Zoom out example image
Probes only (no antigens)
Some cells contain cathepsins
Some cells lack cathepsins
Rare CatS expression
DNA (Hoechst)
CatS (FJD239)
cathepsins (BMV109)
antigens (-)

## Slide 3
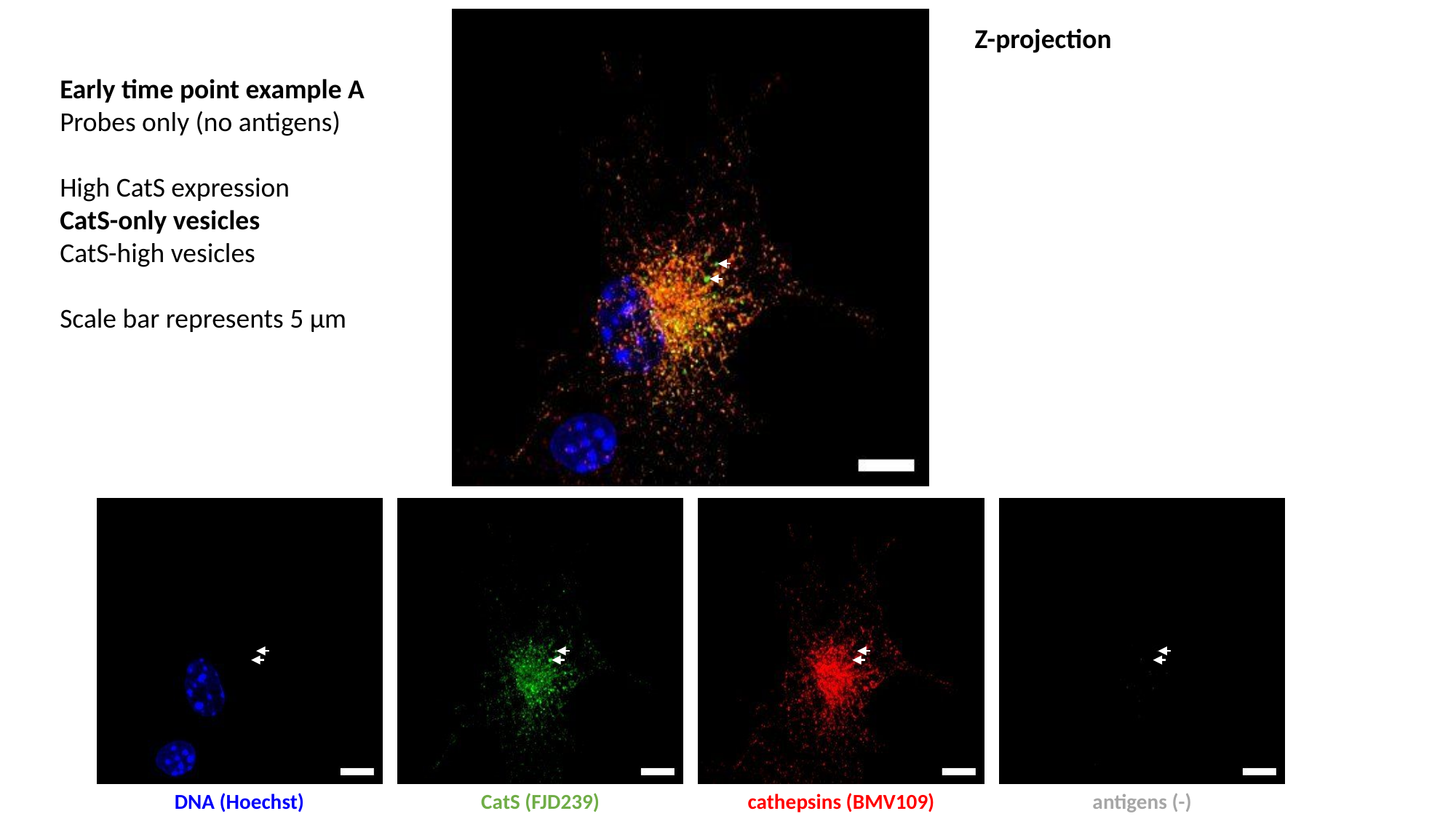

Z-projection
Early time point example A
Probes only (no antigens)
High CatS expression
CatS-only vesicles
CatS-high vesicles
Scale bar represents 5 µm
DNA (Hoechst)
CatS (FJD239)
cathepsins (BMV109)
antigens (-)

## Slide 4
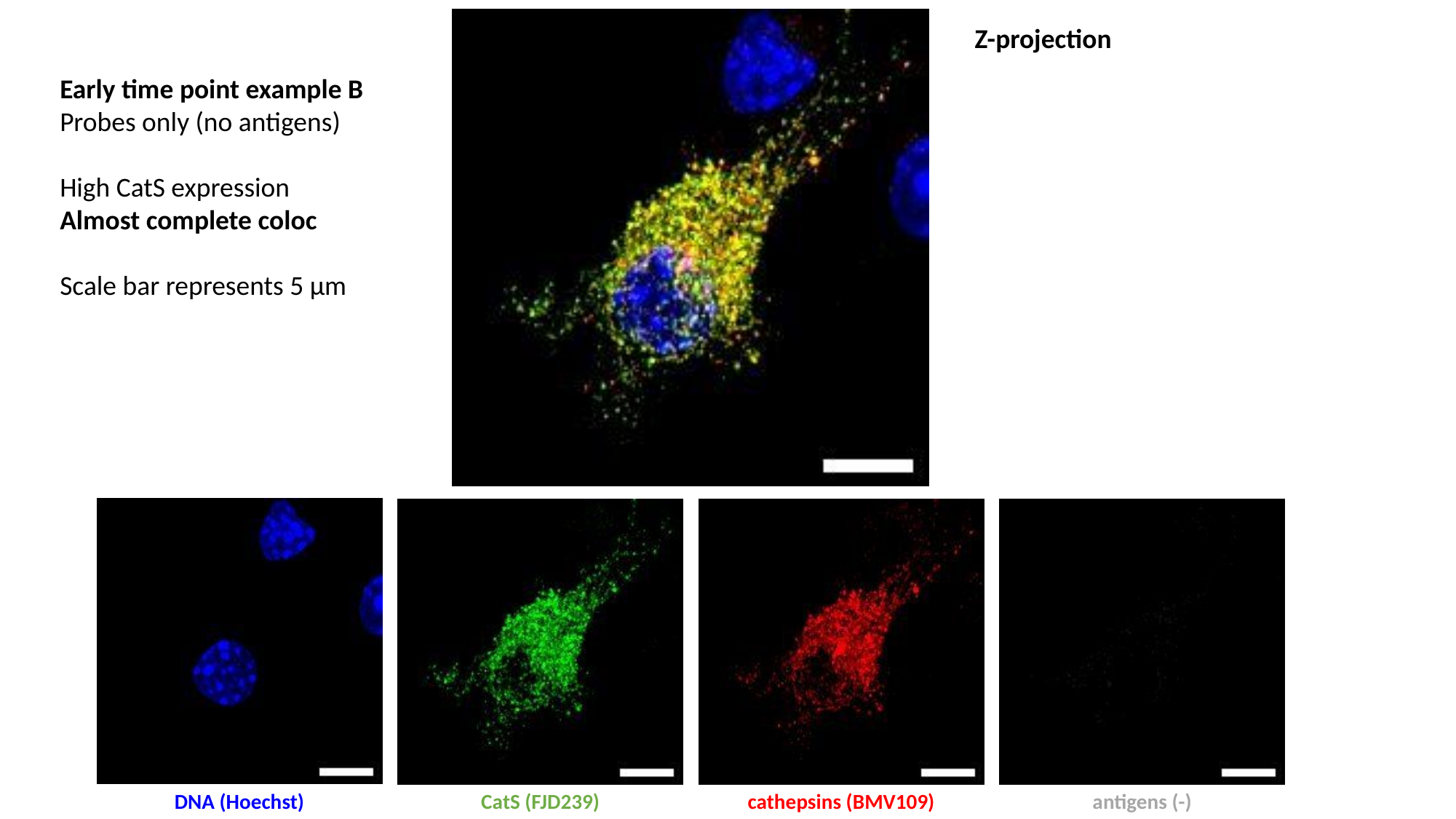

Z-projection
Early time point example B
Probes only (no antigens)
High CatS expression
Almost complete coloc
Scale bar represents 5 µm
DNA (Hoechst)
CatS (FJD239)
cathepsins (BMV109)
antigens (-)

## Slide 5
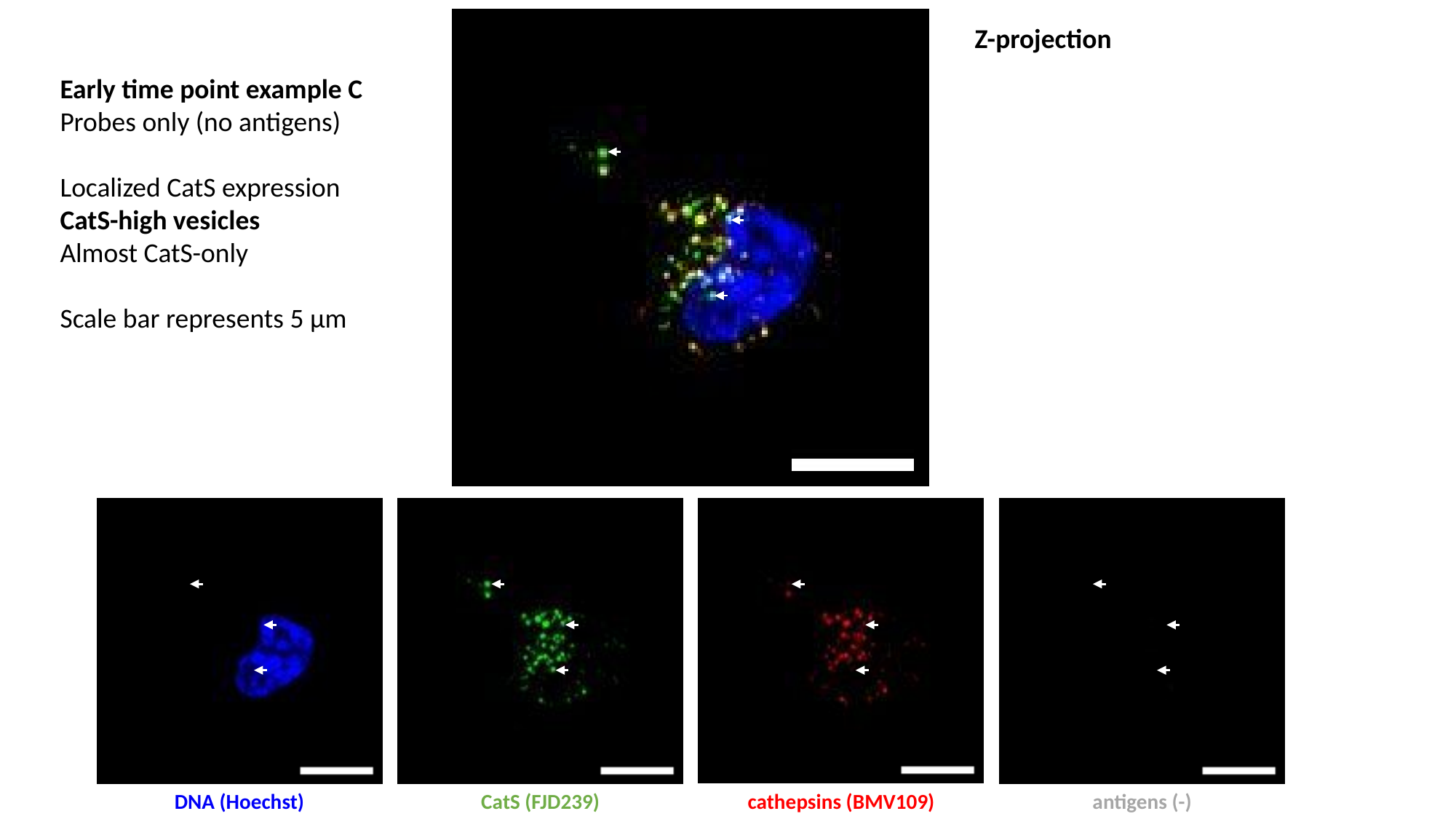

Z-projection
Early time point example C
Probes only (no antigens)
Localized CatS expression
CatS-high vesicles
Almost CatS-only
Scale bar represents 5 µm
DNA (Hoechst)
CatS (FJD239)
cathepsins (BMV109)
antigens (-)

## Slide 6
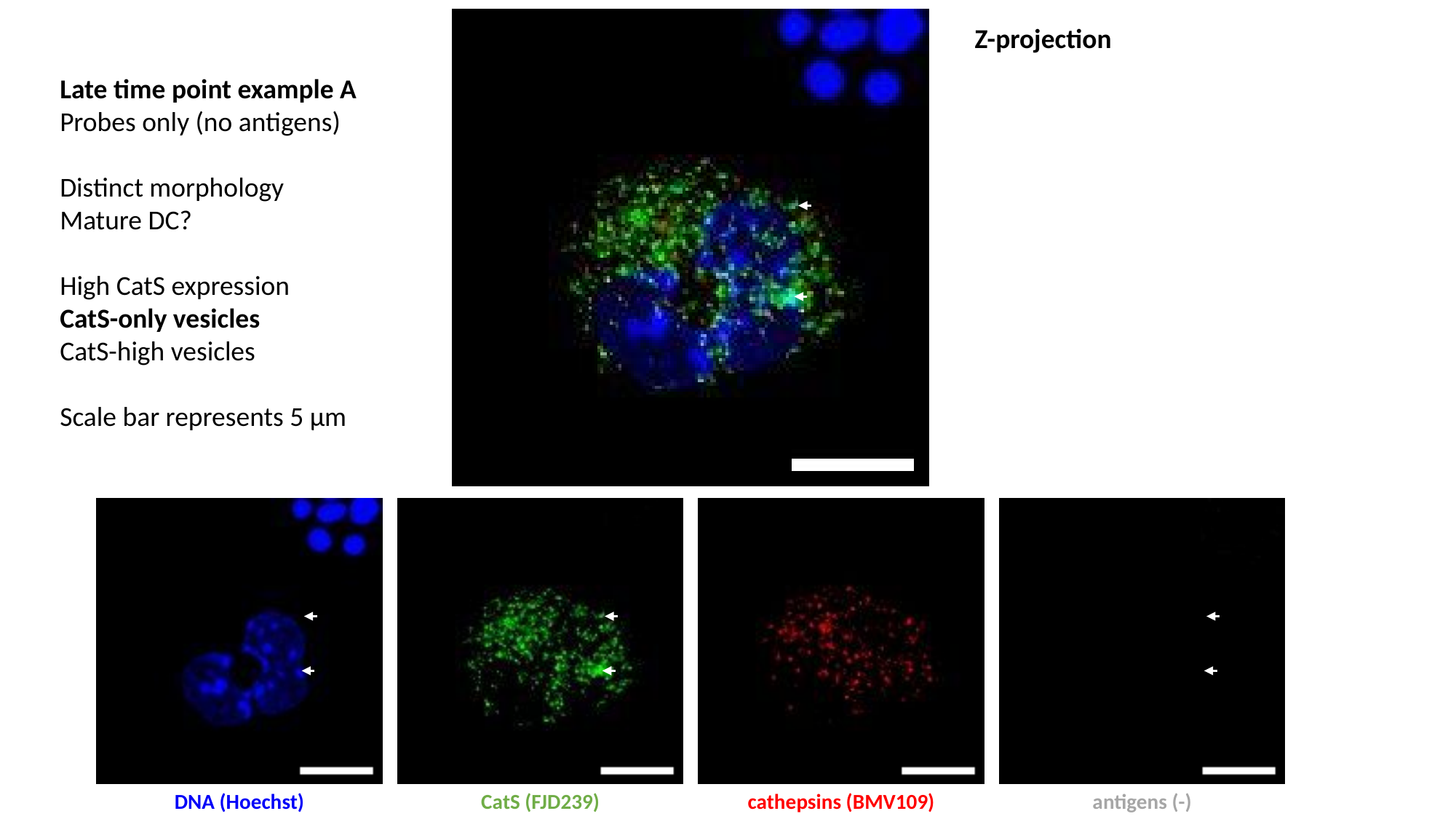

Z-projection
Late time point example A
Probes only (no antigens)
Distinct morphology
Mature DC?
High CatS expression
CatS-only vesicles
CatS-high vesicles
Scale bar represents 5 µm
DNA (Hoechst)
CatS (FJD239)
cathepsins (BMV109)
antigens (-)

## Slide 7
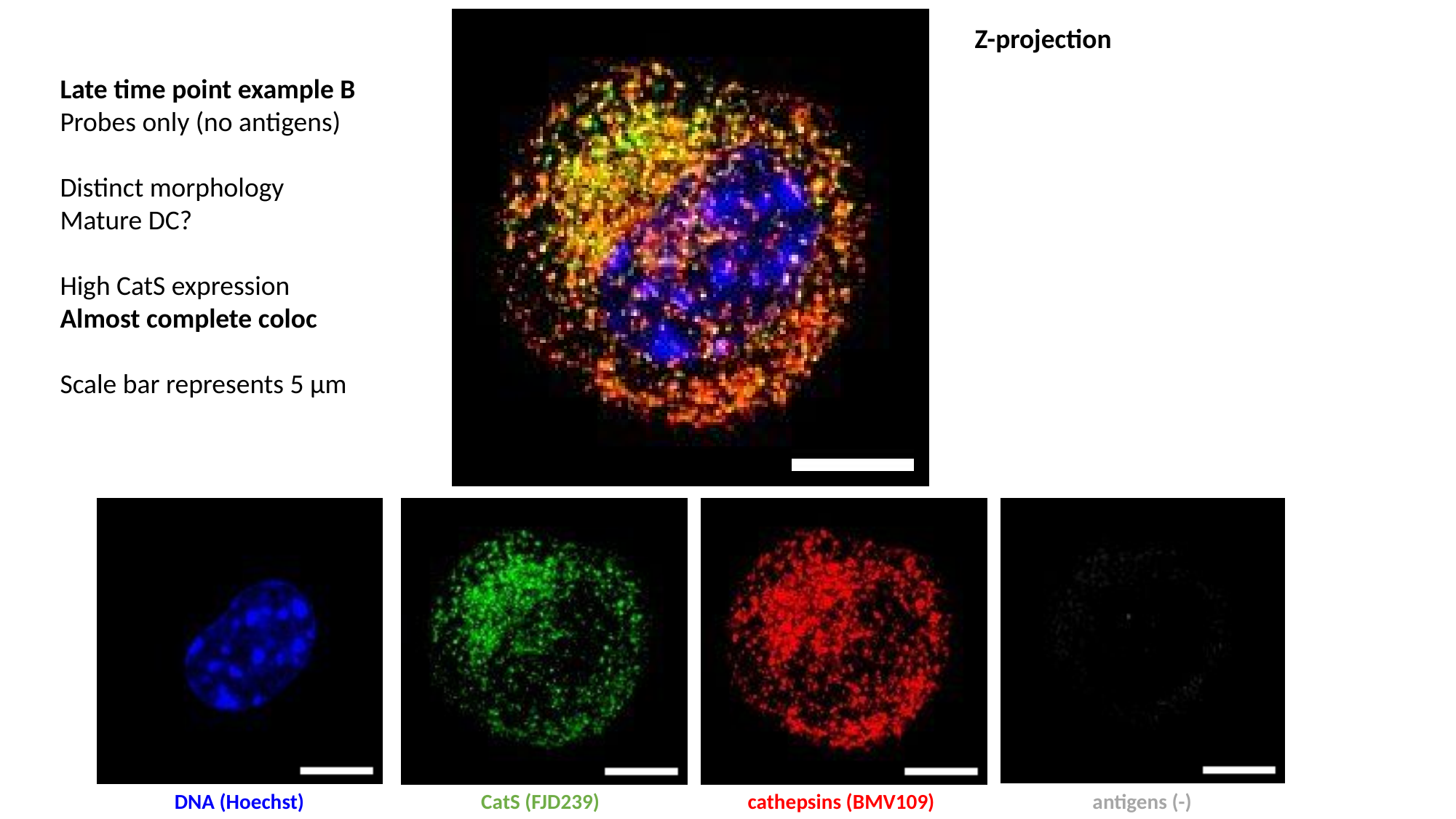

Z-projection
Late time point example B
Probes only (no antigens)
Distinct morphology
Mature DC?
High CatS expression
Almost complete coloc
Scale bar represents 5 µm
DNA (Hoechst)
CatS (FJD239)
cathepsins (BMV109)
antigens (-)

## Slide 8
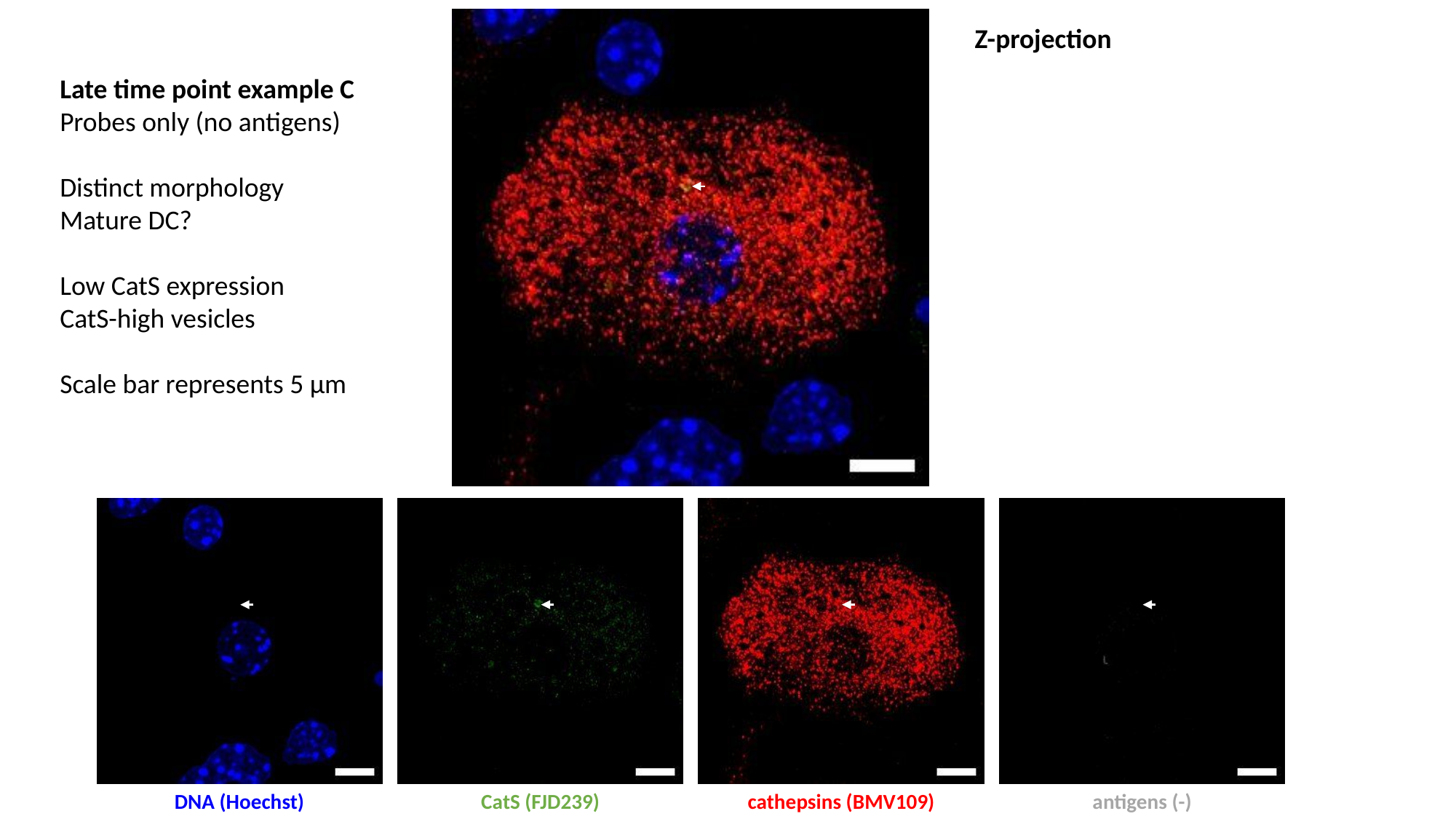

Z-projection
Late time point example C
Probes only (no antigens)
Distinct morphology
Mature DC?
Low CatS expression
CatS-high vesicles
Scale bar represents 5 µm
DNA (Hoechst)
CatS (FJD239)
cathepsins (BMV109)
antigens (-)

## Slide 9
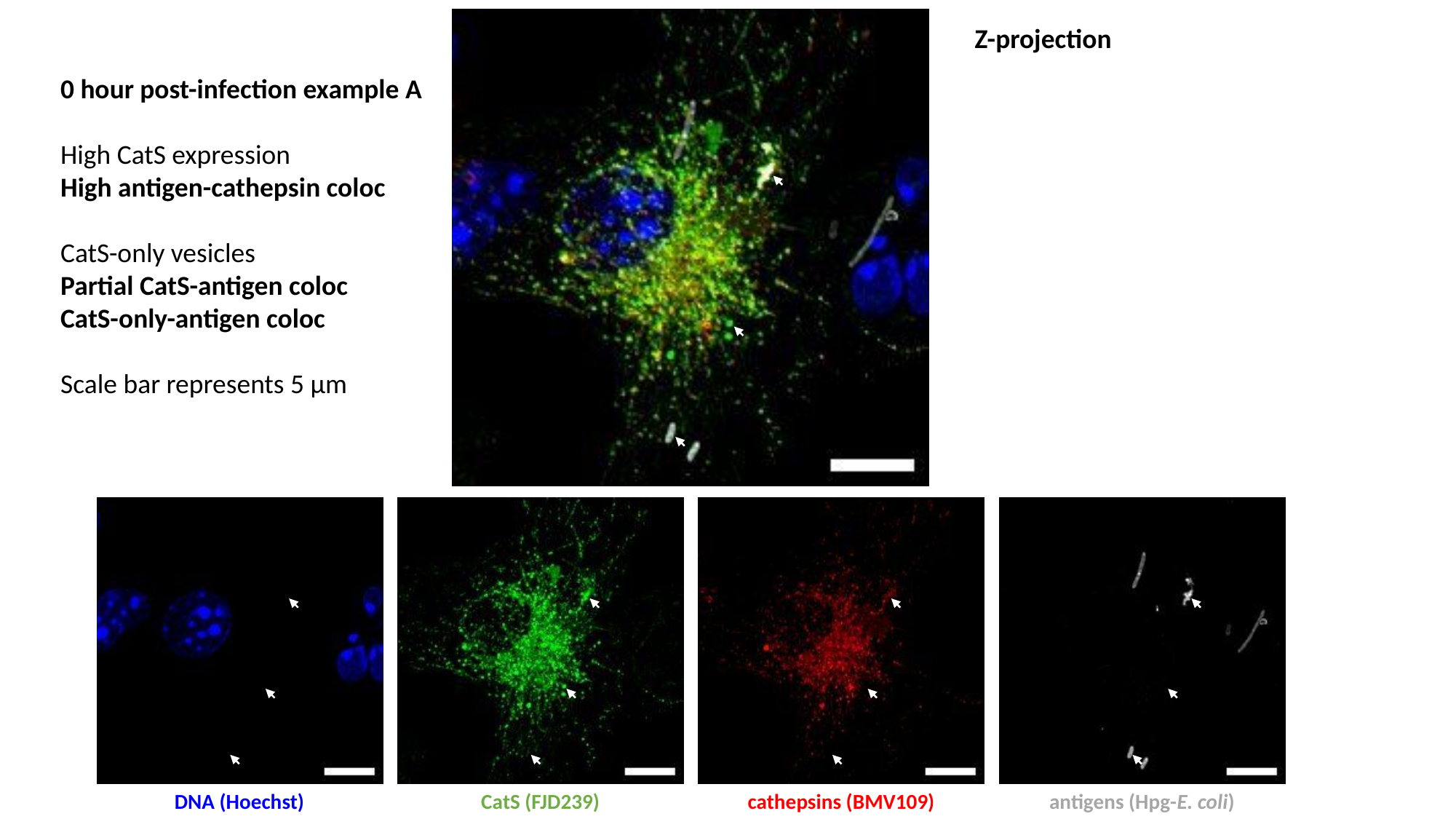

Z-projection
0 hour post-infection example A
High CatS expression
High antigen-cathepsin coloc
CatS-only vesicles
Partial CatS-antigen coloc
CatS-only-antigen coloc
Scale bar represents 5 µm
DNA (Hoechst)
CatS (FJD239)
cathepsins (BMV109)
antigens (Hpg-E. coli)

## Slide 10
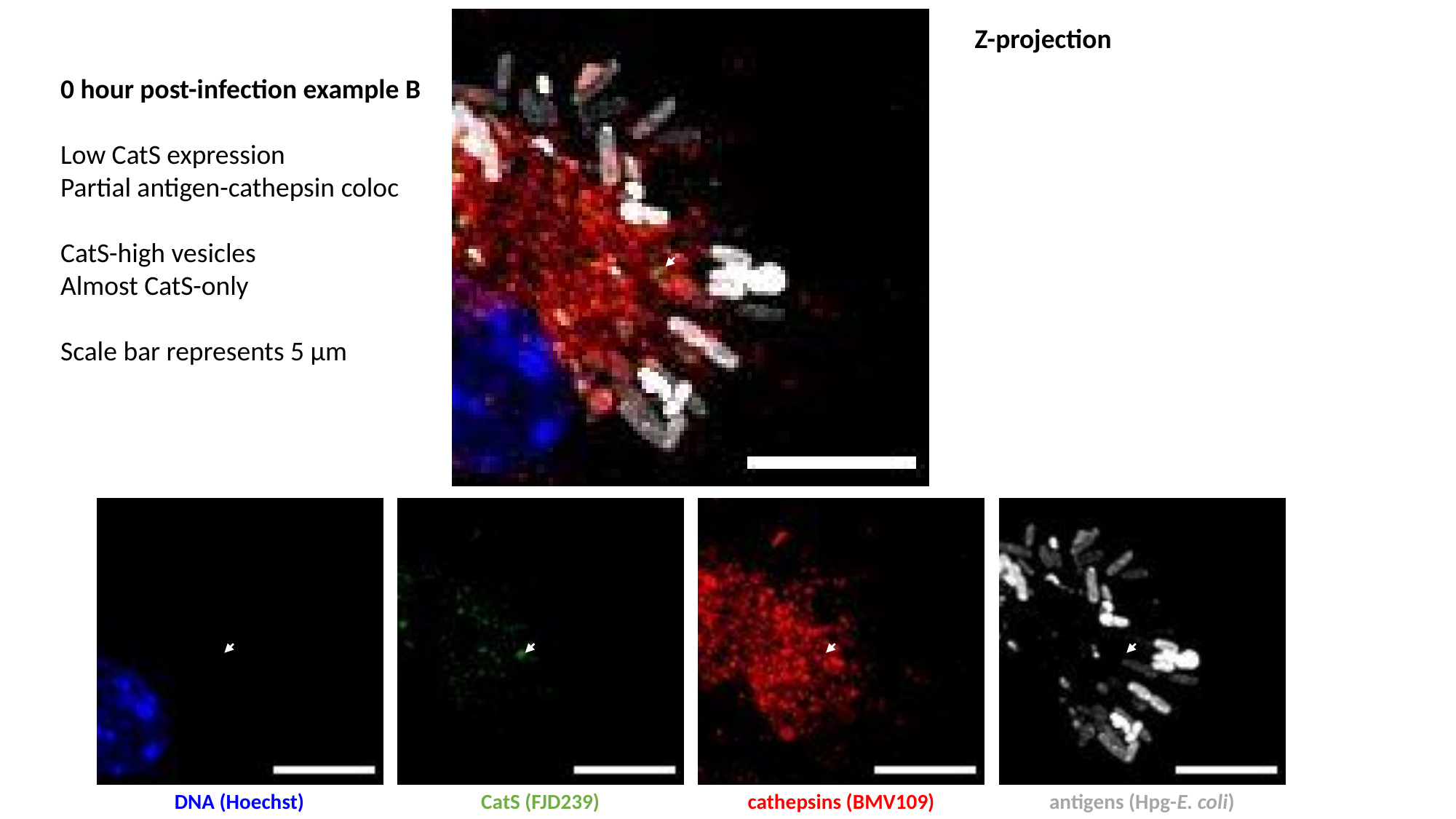

Z-projection
0 hour post-infection example B
Low CatS expression
Partial antigen-cathepsin coloc
CatS-high vesicles
Almost CatS-only
Scale bar represents 5 µm
DNA (Hoechst)
CatS (FJD239)
cathepsins (BMV109)
antigens (Hpg-E. coli)

## Slide 11
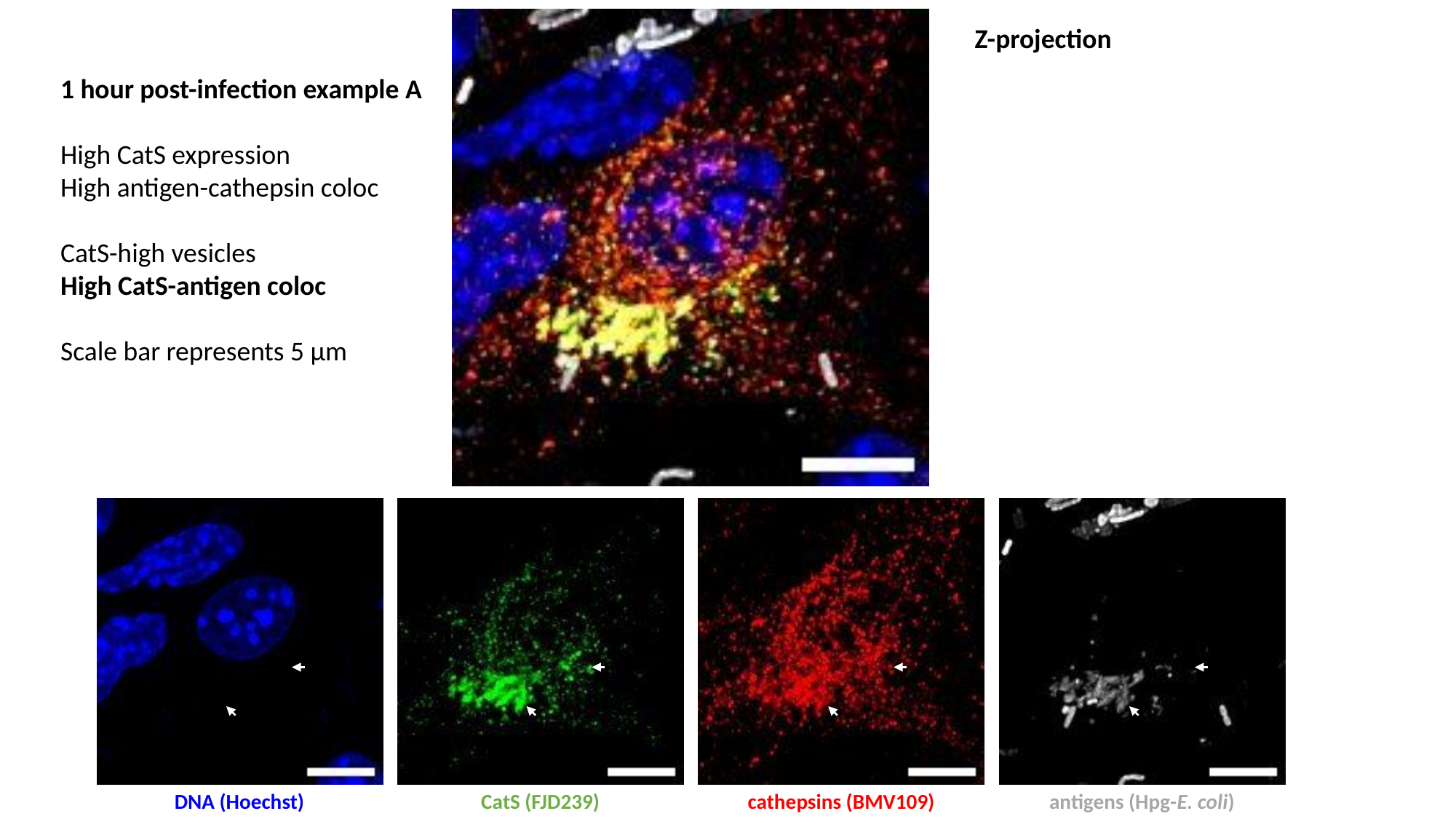

Z-projection
1 hour post-infection example A
High CatS expression
High antigen-cathepsin coloc
CatS-high vesicles
High CatS-antigen coloc
Scale bar represents 5 µm
DNA (Hoechst)
CatS (FJD239)
cathepsins (BMV109)
antigens (Hpg-E. coli)

## Slide 12
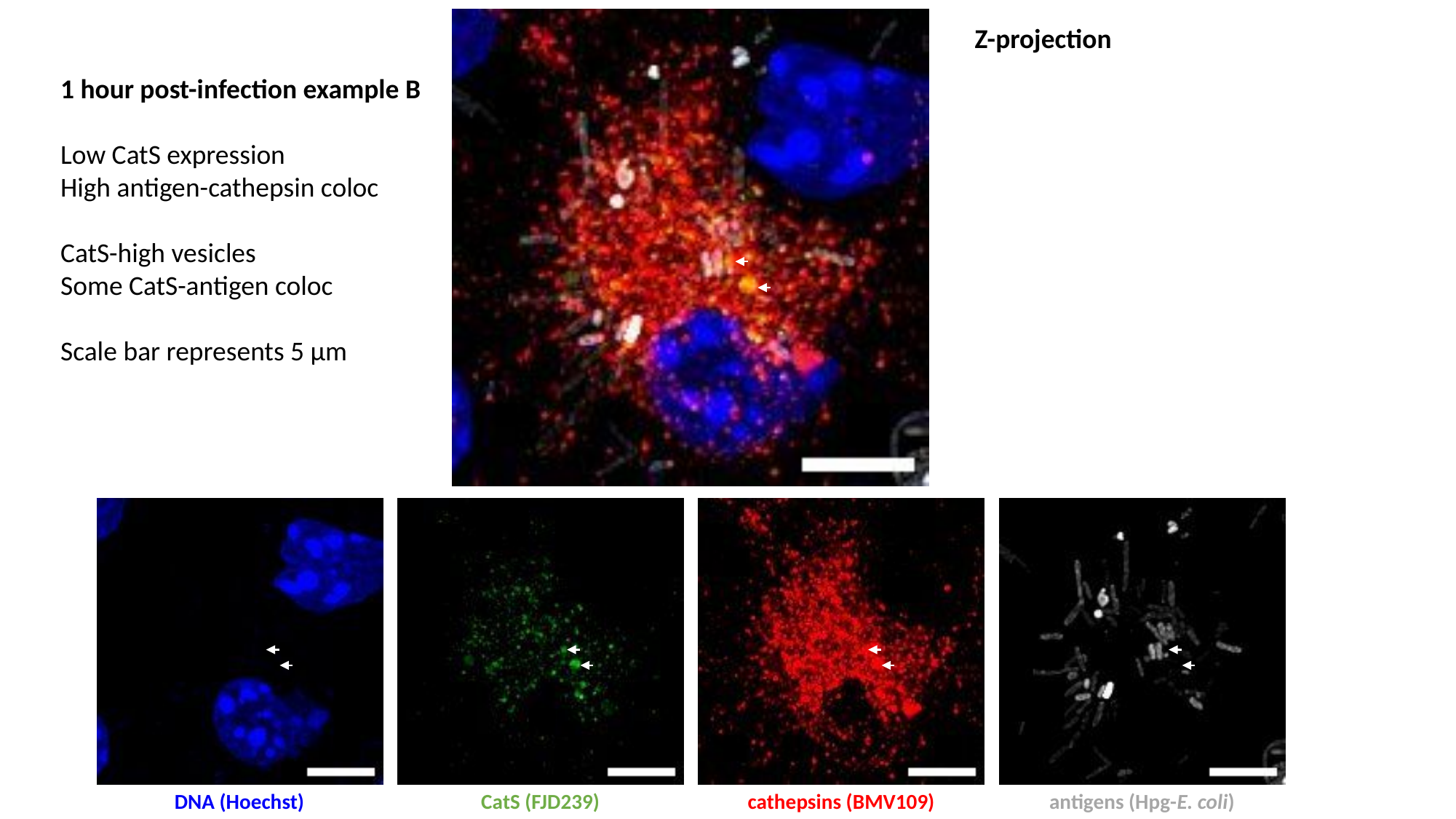

Z-projection
1 hour post-infection example B
Low CatS expression
High antigen-cathepsin coloc
CatS-high vesicles
Some CatS-antigen coloc
Scale bar represents 5 µm
DNA (Hoechst)
CatS (FJD239)
cathepsins (BMV109)
antigens (Hpg-E. coli)

## Slide 13
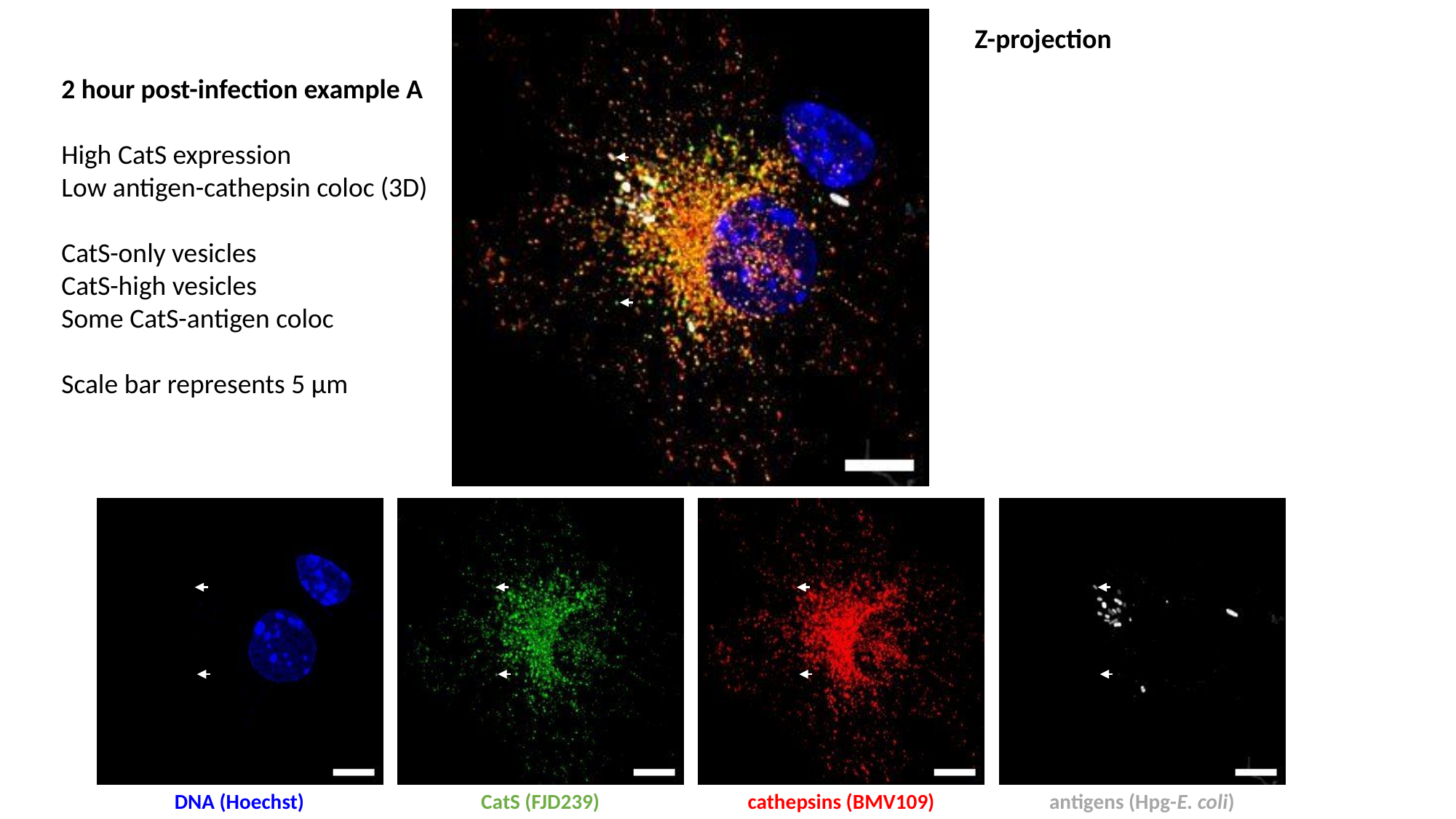

Z-projection
2 hour post-infection example A
High CatS expression
Low antigen-cathepsin coloc (3D)
CatS-only vesicles
CatS-high vesicles
Some CatS-antigen coloc
Scale bar represents 5 µm
DNA (Hoechst)
CatS (FJD239)
cathepsins (BMV109)
antigens (Hpg-E. coli)

## Slide 14
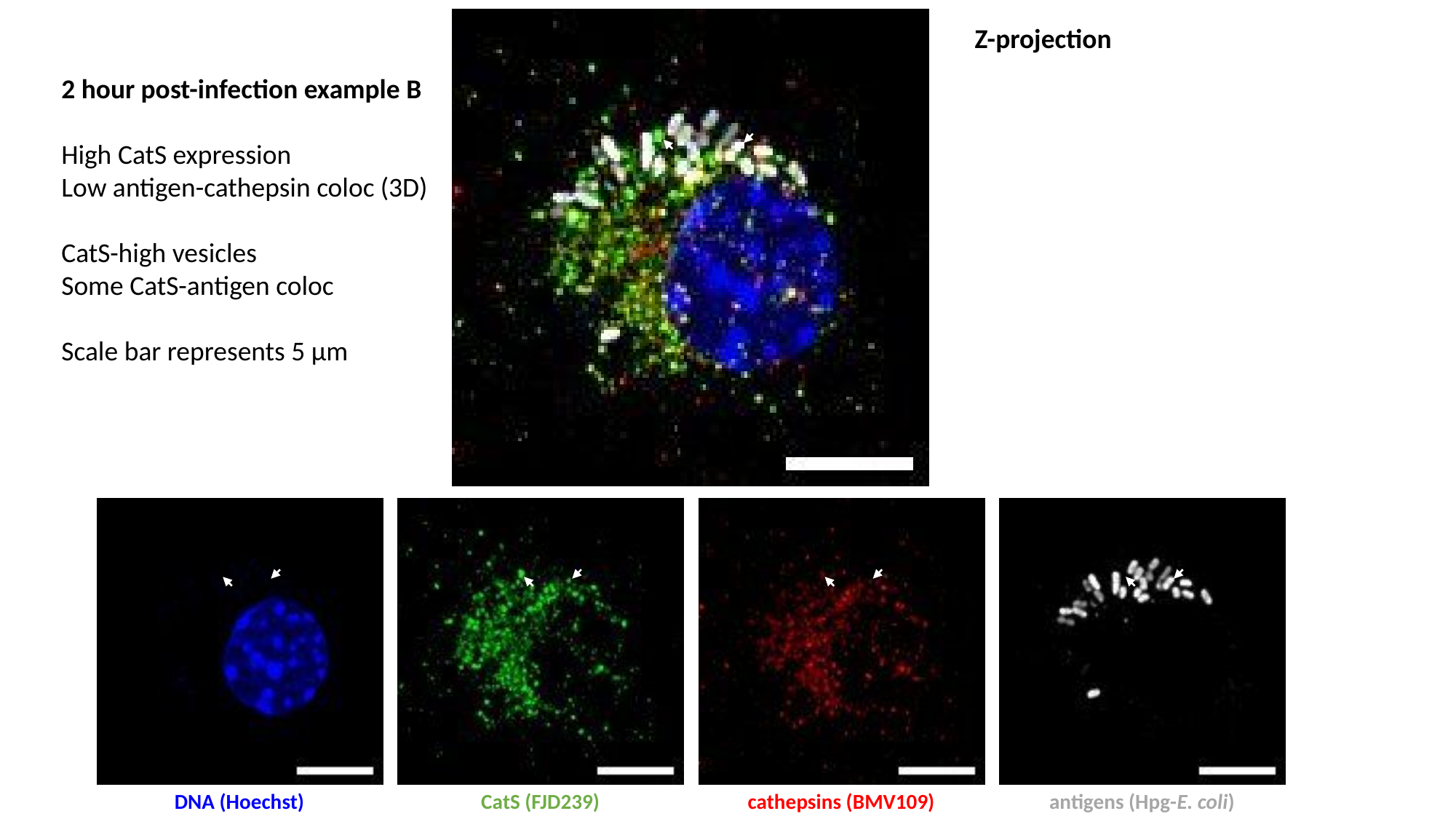

Z-projection
2 hour post-infection example B
High CatS expression
Low antigen-cathepsin coloc (3D)
CatS-high vesicles
Some CatS-antigen coloc
Scale bar represents 5 µm
DNA (Hoechst)
CatS (FJD239)
cathepsins (BMV109)
antigens (Hpg-E. coli)

## Slide 15
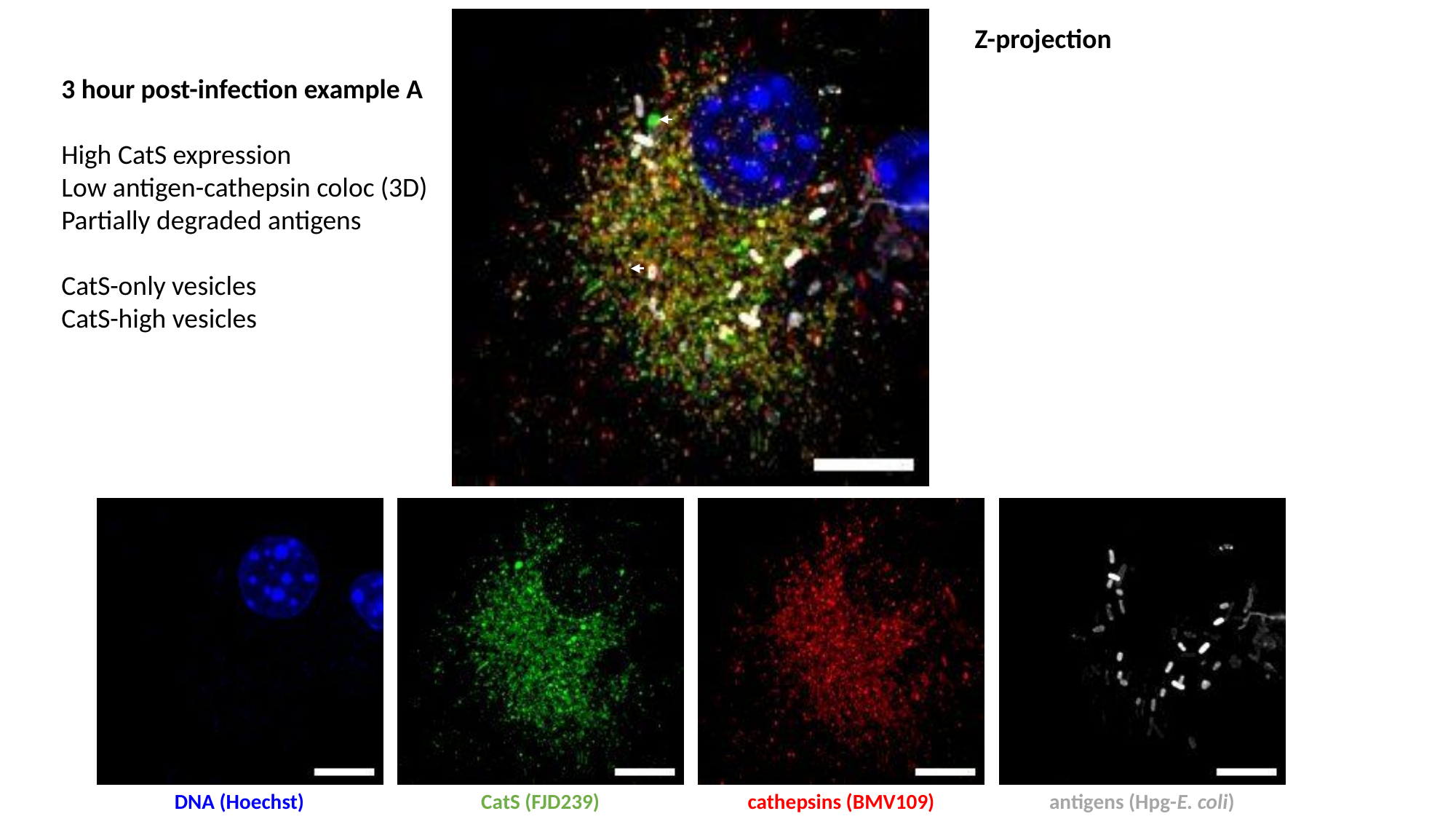

Z-projection
3 hour post-infection example A
High CatS expression
Low antigen-cathepsin coloc (3D)
Partially degraded antigens
CatS-only vesicles
CatS-high vesicles
DNA (Hoechst)
CatS (FJD239)
cathepsins (BMV109)
antigens (Hpg-E. coli)

## Slide 16
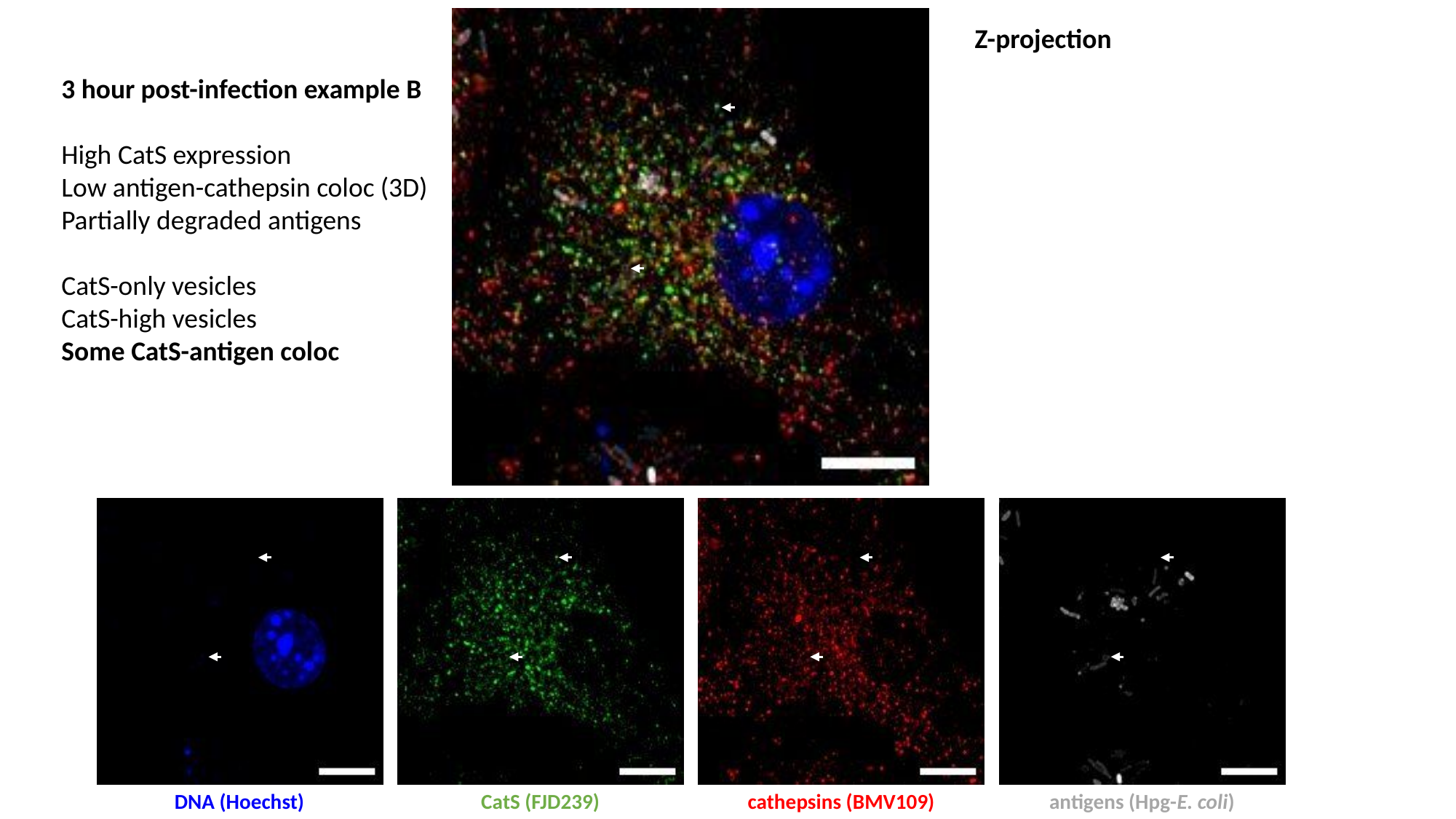

Z-projection
3 hour post-infection example B
High CatS expression
Low antigen-cathepsin coloc (3D)
Partially degraded antigens
CatS-only vesicles
CatS-high vesicles
Some CatS-antigen coloc
DNA (Hoechst)
CatS (FJD239)
cathepsins (BMV109)
antigens (Hpg-E. coli)

## Slide 17
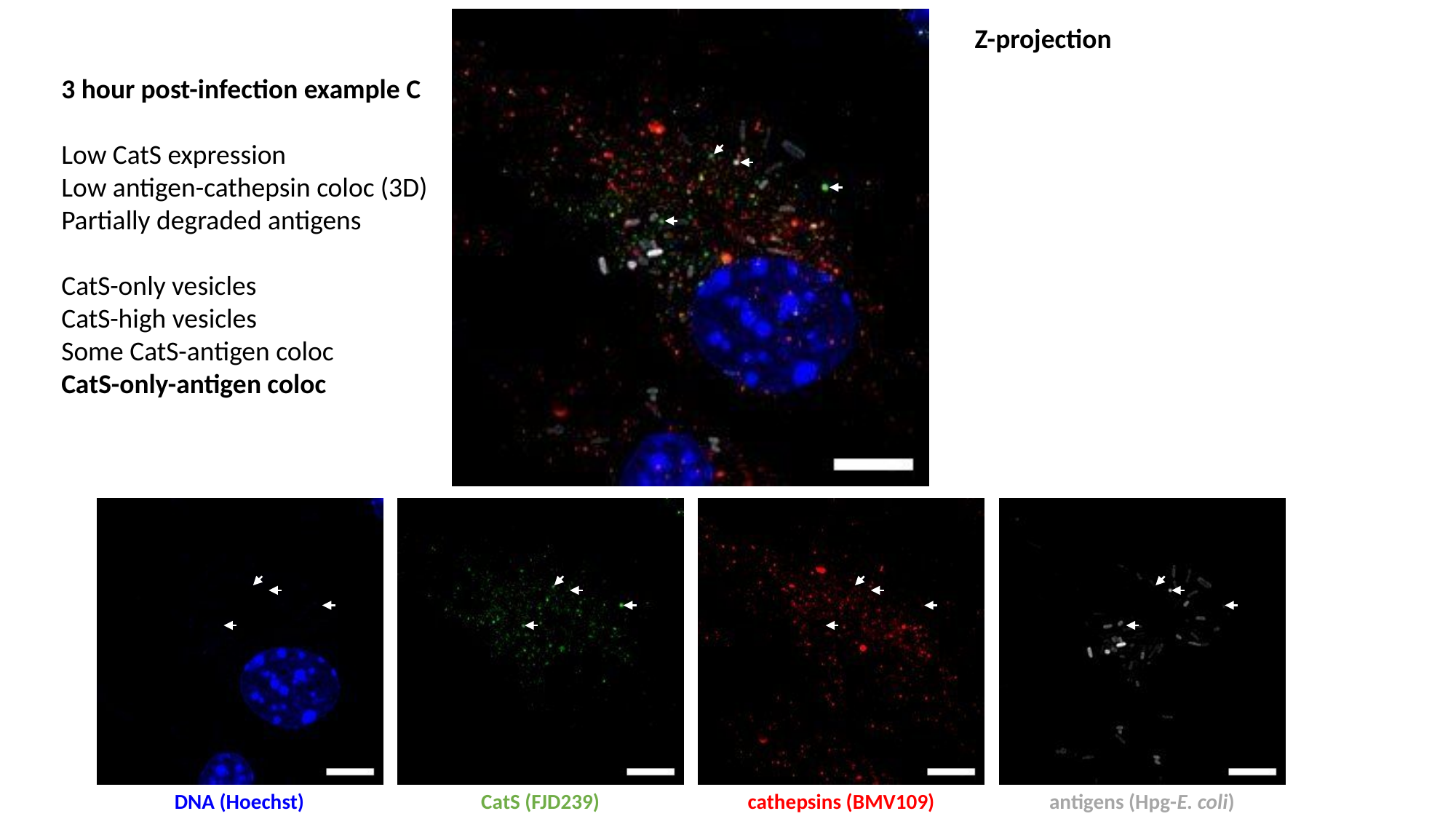

Z-projection
3 hour post-infection example C
Low CatS expression
Low antigen-cathepsin coloc (3D)
Partially degraded antigens
CatS-only vesicles
CatS-high vesicles
Some CatS-antigen coloc
CatS-only-antigen coloc
DNA (Hoechst)
CatS (FJD239)
cathepsins (BMV109)
antigens (Hpg-E. coli)

## Slide 18
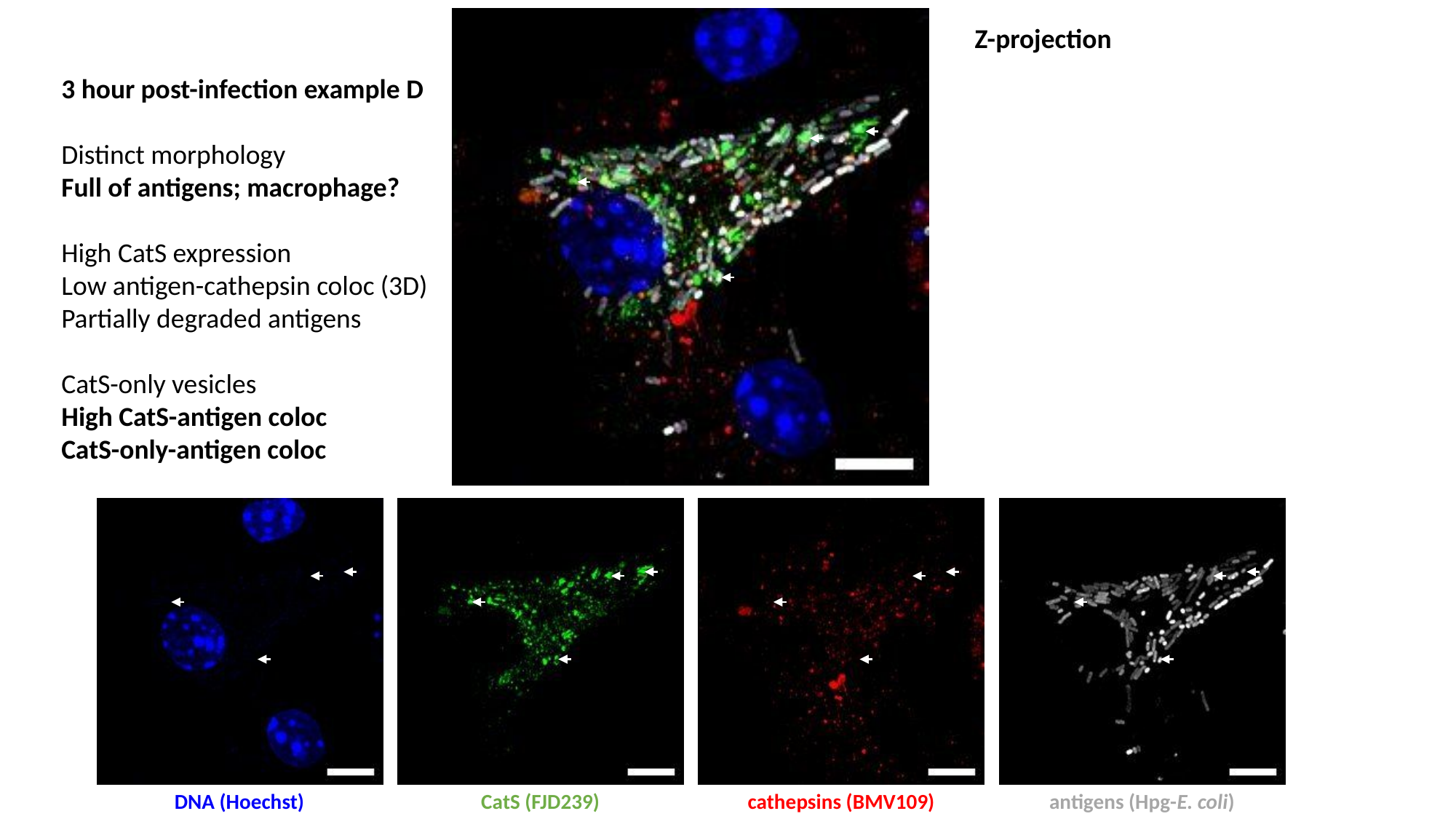

Z-projection
3 hour post-infection example D
Distinct morphology
Full of antigens; macrophage?
High CatS expression
Low antigen-cathepsin coloc (3D)
Partially degraded antigens
CatS-only vesicles
High CatS-antigen coloc
CatS-only-antigen coloc
DNA (Hoechst)
CatS (FJD239)
cathepsins (BMV109)
antigens (Hpg-E. coli)

## Slide 19
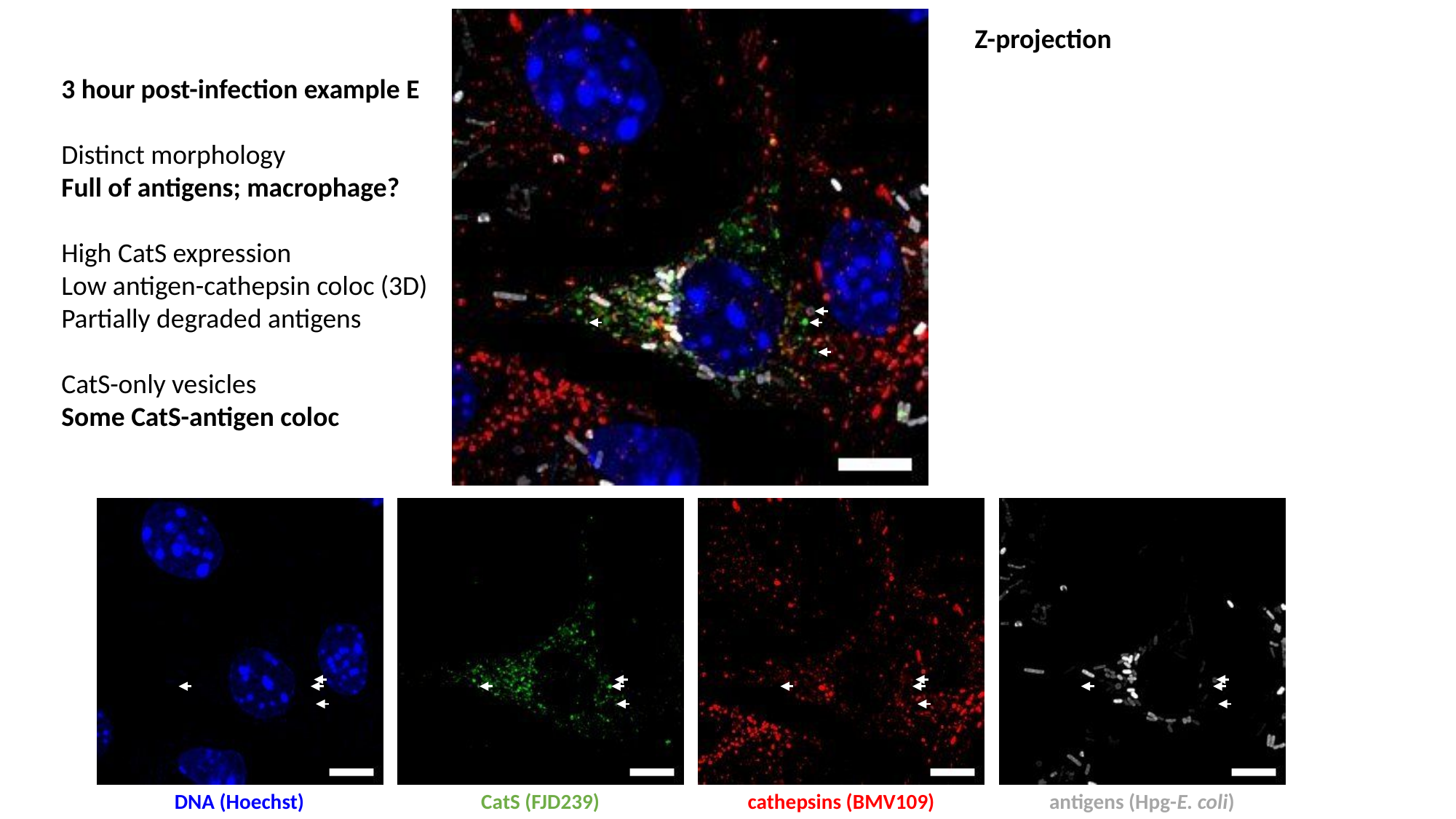

Z-projection
3 hour post-infection example E
Distinct morphology
Full of antigens; macrophage?
High CatS expression
Low antigen-cathepsin coloc (3D)
Partially degraded antigens
CatS-only vesicles
Some CatS-antigen coloc
DNA (Hoechst)
CatS (FJD239)
cathepsins (BMV109)
antigens (Hpg-E. coli)
